# Sulfated chitosan mitigates acute lung injury induced bone loss via immunoregulation

**DOI:** 10.1038/s41413-025-00475-4

**Published:** 2026-02-05

**Authors:** Yongxian Liu, Luli Ji, Fuwei Zhu, Jiaze Yu, Dongao Huang, Jingyuan Cui, Xiaogang Wang, Jing Wang, Changsheng Liu

**Affiliations:** 1 The State Key Laboratory of Bioreactor Engineering, East China University of Science and Technology, Shanghai, China; 2 Engineering Research Center for Biomedical Materials of the Ministry of Education, East China University of Science and Technology, Shanghai, China; 3 Frontiers Science Center for Materiobiology and Dynamic Chemistry, East China University of Science and Technology, Shanghai, China; 4 Key Laboratory for Ultrafine Materials of Ministry of Education, East China University of Science and Technology, Shanghai, China

**Keywords:** Bone

## Abstract

Respiratory inflammatory diseases disrupt bone metabolism and cause pathological bone loss. The lung-bone axis is established in chronic diseases like asthma and cystic fibrosis but is less studied in acute lung injury (ALI), recently implicated in COVID-19-induced bone loss. This study examined the effects of LPS-induced ALI on bone phenotype and explored the role of 2-N, 6-O sulfated chitosan (26SCS) in mitigating pneumonia-induced bone loss via inflammatory response modulation. Our findings show that 26SCS effectively reaches bone tissue after oral administration. It promotes macrophage polarization to the M2 phenotype, alleviating immune cascade reactions and inhibiting osteoclast-mediated bone resorption. Increased M2 macrophages support type H vessel formation, enhancing inflammatory bone vascularization. These effects foster a favorable osteogenic microenvironment and mitigate ALI-induced bone loss. While dexamethasone is effective in reducing inflammation, it can aggravate ALI-induced bone loss. Our research offers a therapeutic strategy targeting the lung-bone axis for inflammation-induced bone loss.

## Introduction

Since its emergence in 2019, the Severe Acute Respiratory Syndrome Coronavirus 2 (SARS-CoV-2), responsible for COVID-19, has placed immense strain on global healthcare systems and economies.^1^ One of the most severe complications of COVID-19 is acute lung injury, particularly severe acute respiratory distress syndrome, which presents a significant threat to life and is associated with a high mortality rate.^2^ In addition to respiratory issues, COVID-19 patients often face a range of complications, including anosmia, myasthenia, thrombosis-induced inflammation, and immune dysregulation.^3–5^ Post-COVID patients have also reported symptoms such as bone pain and a burning sensation.^6^ The lung-bone axis, which links pulmonary inflammatory diseases with bone disorders like bone loss and osteoporosis, has become increasingly recognized. Chronic pulmonary inflammatory diseases, including chronic obstructive pulmonary disease, asthma, and cystic fibrosis, have been associated with bone loss.^7,8^ Yang et al. showed that SARS-CoV-2 does not directly infect bones but influences bone metabolism through the release of pro-inflammatory factors, ultimately leading to bone loss.^9^ These inflammatory factors from the lungs interact with the skeletal system via the circulatory system.^10^ However, the precise mechanisms remain unclear, and effective therapeutic strategies are still lacking.

Bone is a dynamic tissue that continuously balances regeneration and resorption, a process regulated by factors shared between the skeletal and immune systems.^11^ Inflammation disrupts this balance by overactivating cytokines such as interleukin-1 (IL-1), interleukin-6 (IL-6), and tumor necrosis factor-alpha (TNF-α), which enhance osteoclast activity and suppress osteoblast function.^12,13^ Pro-inflammatory cytokines drive osteoclast differentiation through receptor activator of nuclear factor-kappa B ligand (RANKL) and macrophage colony-stimulating factor (M-CSF), with TNF-α promoting monocyte-to-osteoclast precursor differentiation via its p55 receptor and activating pathways like spleen tyrosine kinase (SYK) to amplify resorption.^14–18^ Simultaneously, inflammatory factors induce glucocorticoid release through the hypothalamic-pituitary-adrenal (HPA) axis, further exacerbating bone loss, and suppress osteoblast-mediated bone formation by upregulating Dickkopf-1 (DKK1) and sclerostin, which inhibit Wnt/β-catenin signaling.^19–21^ While anti-inflammatory therapies can reduce the production of inflammatory factors, inflammation-induced bone loss may persist.^22,23^ Macrophages, key mediators of inflammatory bone loss, exhibit plasticity and polarize into pro-inflammatory M1 or anti-inflammatory M2 phenotypes.^24^ M1 macrophages, stimulated by TNF-α, lipopolysaccharide (LPS), and interferon-gamma (IFN-γ), secrete IL-1β and IL-6, intensifying inflammation and promoting osteoclast activity.^25,26^ In contrast, M2 macrophages release bone morphogenetic protein 2 (BMP-2), transforming growth factor-beta (TGF-β), and vascular endothelial growth factor (VEGF), activating Wnt/Lrp5 signaling to support bone regeneration and angiogenesis, creating a microenvironment conducive to repair.^27,28^ These interactions highlight the intricate link between inflammation, macrophage polarization, and bone metabolism.

Glucocorticoids (GCs) have proven highly effective in managing inflammation, offering relief in conditions such as cystic fibrosis and inflammatory bowel disease.^29^ However, prolonged or high-dose use of GCs is known to cause bone loss via activated osteoclasts.^30,31^ Recent research has highlighted that dexamethasone (DEX) aggravates bone loss by impairing vascular health and disrupting local nutrient supply.^32^ Therefore, current GC medications can alleviate inflammation but pose a threat to bone health. Type H blood vessels, characterized by high permeability and oxygenation, play a crucial role in maintaining bone metabolism.^33^ Significant loss of type H vessels has been observed in both age-related bone loss and ovariectomy models.^34^ Studies have shown that Type H vessel formation is promoted by M2 macrophages in lower limb ischemia models through the secretion of VEGF.^35^ Therefore, regulating inflammation, along with a healthy bone microenvironment via type H vessel maintenance, represent an ideal strategy for treating inflammatory diseases.

Sulfated glycosaminoglycans (GAGs) are widely present in mammalian tissues and are key components of the extracellular matrix, playing an essential role in regulating angiogenesis, cancer response, and various other biological events.^36–38^ However, their main limitation lies in the variability of their chemical structure, which is influenced not only by animal sources but also by individual physiological and pathological conditions.^39^ As a solution, the development of semi-synthetic GAGs through regional selective sulfation of widely available polysaccharides may provide an effective alternative. The high reactivity of functional groups, such as primary hydroxyl or amino groups along the polysaccharide chain, offers opportunities for selective modification.^40^ Our group successfully achieved the sulfonation of chitosan, resulting in structurally well-defined 2-N, 6-O sulfated chitosan (26SCS).^41^ Previous studies have shown that 26SCS exerts immunoregulatory functions through promoting macrophage polarization toward an anti-inflammatory phenotype and facilitates the ingrowth of type H vessels.^35,42^ These findings suggest that 26SCS may play a positive role in mitigating inflammation-induced bone loss.

Here we established an LPS-induced mouse ALI model, which resulted in a marked increase in the inflammatory cytokines including IL-1β, TNF-α, and IL-6 both at the lesion site and in the serum. This inflammatory response triggered a cascade in the femur, leading to macrophage M1 polarization, which in turn secreted more pro-inflammatory cytokines, driving osteoclast activation and bone loss. Oral administration of 26SCS significantly reduced the levels of these inflammatory cytokines in the lungs, serum, and bone marrow, demonstrating its effective anti-inflammatory properties. Dexamethasone effectively suppresses inflammation but impairs type H vessels, leading to increased bone loss. Mechanistic studies revealed that 26SCS promotes macrophage polarization to the M2 anti-inflammatory phenotype, which inhibits osteoclast activity and enhances the formation of type H blood vessels. These combined effects contribute to the creation of a favorable bone microenvironment, ultimately ameliorating bone loss.

## Results

### LPS-induced ALI leads to bone loss

**Fig. 1:**
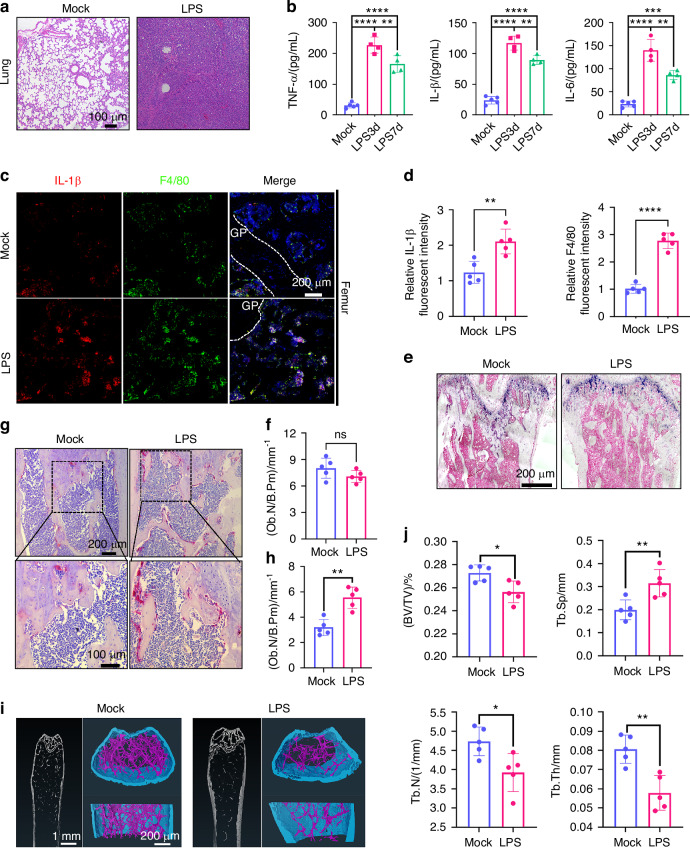
ALI induced by LPS leads to systemic inflammation and bone loss in mice. **a** Representative H&E-stained lung tissue images from mice on day 3 after LPS induction. **b** Levels of IL-1β, TNF-α, and IL-6 in the serum of ALI mice measured by ELISA at various time points (*n* = 5). **c** Representative multicolor immunofluorescence staining of F4/80 and IL-1β in the distal metaphysis of the femur on day 7 after LPS induction. DAPI was used for nuclear counterstaining. **d** Quantitative analysis of IL-1β and F4/80 immunofluorescence intensity in C (*n* = 5). **e** Representative ALP staining images of femoral trabecular bone showing osteoblast activity under mock and LPS-treated conditions. **f** Histomorphometric analysis of osteoblast numbers per bone perimeter (Ob.N/B.Pm) in femoral sections (*n* = 5). **g** Representative TRAP staining of the distal metaphysis of the femur with high-magnification views of the boxed regions. **h** Quantitative analysis of TRAP^+^ osteoclast numbers in the distal metaphysis of the femur (*n* = 5). **i** Representative μCT images of the femur on day 30 after LPS induction. **j** Bone parameter quantification based on μCT analysis, including trabecular thickness (Tb.Th), bone volume fraction (BV/TV), trabecular number (Tb.N), and trabecular separation (Tb.Sp) (*n* = 5). Statistical analysis: Data are presented as mean ± standard deviation (SD). *P < 0.05, **P < 0.01, ***P < 0.005, ****P < 0.001

To investigate the impact of pneumonia on bone, we established an acute lung injury model in C57 mice using LPS. Significant disease features were observed on day 3 post-infection, with notable recovery by day 7. On day 3, lung tissues were collected and subjected to hematoxylin and eosin (H&E) staining, revealing extensive inflammatory cell infiltration, thickened alveolar walls, and disrupted alveolar spaces in the model group compared to normal mice (Fig. 1a). Serum samples were also analyzed using enzyme-linked immunosorbent assay (ELISA) to quantify inflammatory cytokines, including interleukin-1 beta (IL-1β), tumor necrosis factor-alpha (TNF-α), and interleukin-6 (IL-6) (Fig. 1b). The results indicated a significant increase in these cytokine levels in the LPS group. While the immune response led to partial downregulation of these cytokines by day 7, their levels remained elevated compared to the mock group.

To clarify the impact of persistent pneumonia on the bone immune environment, we analyzed the inflammatory levels in the femurs of mice on day 7 using immunohistochemistry (IHC) and immunofluorescence (IF) staining (Figs. 1c and S1a). The IHC staining showed a significant increase in the levels of IL-1β, TNF-α, and IL-6 in the femur (Fig. S1b). Additionally, the IF results revealed an increased number of macrophages and higher expression of IL-1β in the femurs of mice with acute lung injury (Fig. 1d). The colocalized expression of the macrophage marker F4/80 and IL-1β suggests that circulating inflammatory factors trigger a response in the bone immune microenvironment, initiating an inflammatory cascade in the bone. These findings indicate that during acute lung injury, inflammatory cytokines enter the circulatory system, reach the skeletal system, and stimulate macrophages in the bone, exacerbating the inflammatory response and leading to increased cytokine expression in the femoral microenvironment. This cytokine overexpression may further activate osteoclasts.

To assess the impact of this inflammatory response on bone tissue, we performed tartrate-resistant acid phosphatase (TRAP) staining (Fig. 1g), alkaline phosphatase (ALP) staining (Fig. 1e, f), and HE staining on the bone tissue of mice on day 7 (Fig. S1c). TRAP staining revealed a significant increase in the number of TRAP^+^ osteoclasts in the trabecular bone of the distal femoral metaphysis in LPS-treated mice, with osteoclast numbers approximately 1.8 times higher than in the control group (Fig. 1h). ALP staining showed that LPS treatment also led to a notable reduction in osteoblasts. These results suggest that inflammatory cytokines primarily influenced osteoclast activity. Consequently, our subsequent focus was on studying osteoclasts. H&E staining further revealed bone loss in LPS-treated mice (Fig. S1c). To better understand the long-term effects of LPS-induced lung infection on bone, femurs were isolated on day 30 and analyzed using three-dimensional micro-computed tomography (micro-CT) to assess bone-related parameters (Fig. 1i). Compared to the mock group, LPS-treated mice exhibited a significant decrease in bone volume fraction (BV/TV), trabecular number (Tb.N), and trabecular thickness (Tb.Th), along with an increase in trabecular separation (Tb.Sp), all of which contributed to severe bone loss (Fig. 1j). Taken together, LPS-induced ALI caused an upregulation of inflammatory factors in the femur and excessive osteoclast activity, leading to significant bone loss.

### 26SCS alleviates ALI-induced bone loss

**Fig. 2:**
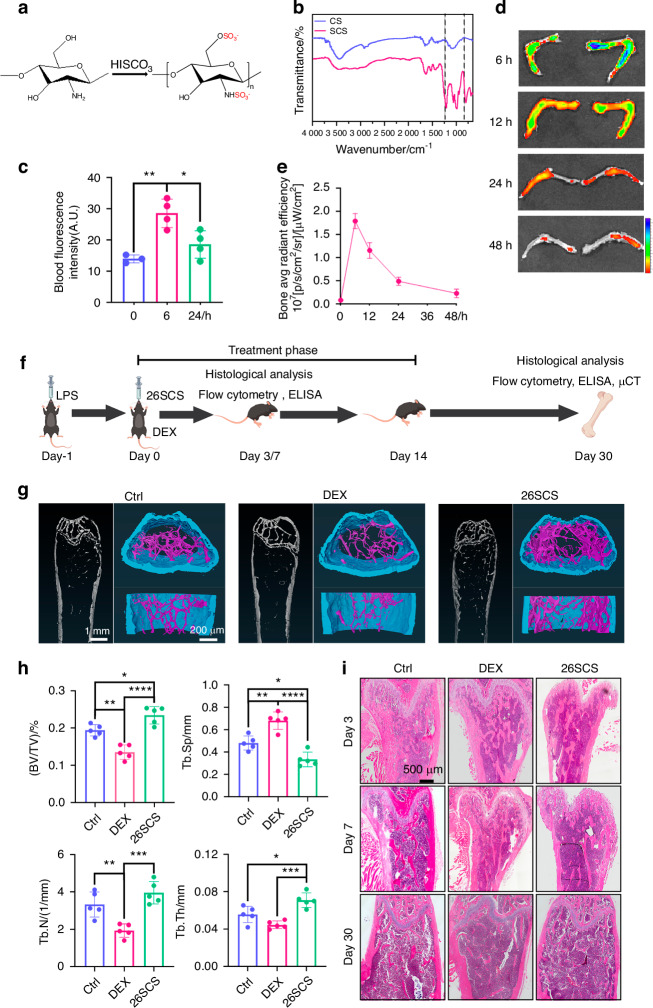
26SCS improves bone loss caused by LPS-induced acute lung injury (ALI). **a** Schematic illustration of 26SCS synthesis. **b** Fourier-transform infrared spectroscopy (FTIR) characterization of chitosan (CS) and 26SCS, showing characteristic peaks at 1 200 cm⁻¹ and 800 cm⁻¹ corresponding to O = S = O and C-O-S groups, respectively. **c** Quantification of blood fluorescence intensity at 6 and 24 h respectively after oral administration of fluorescence-labeled 26SCS (*n* = 3 for 0 h, *n* = 4 for 6 h and 24 h). **d** Ex vivo fluorescence imaging of femurs harvested at different time points (6, 12, 24, and 48 h) after oral administration of fluorescence-labeled 26SCS. **e** Time course of fluorescence intensity in bone tissue after oral administration (*n* = 3). **f** Experimental design schematic. Mice were treated with LPS (day -1) and subsequently treated with PBS (control), dexamethasone (DEX), or 26SCS (day 0). Samples were collected on days 3, 7, and 30 for indicated analysis. **g** Representative μCT images of femurs on day 30. **h** Quantitative analysis of bone parameters based on μCT, including bone volume fraction (BV/TV), trabecular separation (Tb.Sp), trabecular thickness (Tb.Th), and trabecular number (Tb.N) (*n* = 5). **i** Representative H&E-stained images of the distal femoral metaphysis on days 3, 7, and 30. Statistical analysis: Data are presented as mean ± standard deviation (SD). *P < 0.05, **P < 0.01, ***P < 0.005, ****P < 0.001

To investigate the effect of 26SCS on modulation of ALI-triggered systematic inflammation, we synthesized 26SCS as previously reported.^41^ Fourier transform infrared spectroscopy (FTIR) analysis revealed peaks at 1 200 cm⁻¹ and 800 cm⁻¹, corresponding to O = S = O and C-O-S functional groups, respectively, suggesting the successful sulfonation of chitosan (Fig. 2a, b). Elemental analysis showed that synthesized 26SCS contained a sulfur content of 13% ± 0.1% (Fig. S2a). Oral administration was chosen due to its advantages of being non-invasive, allowing for easier and more frequent dosing, and enhancing patient compliance in potential clinical applications. To determine whether oral administration of 26SCS could reach the bone area, we used FITC-labeled 26SCS for oral gavage with the dosage of 5 mg/kg. After 6 h, we observed a significant increase in fluorescence intensity in the bloodstream. However, at 24 h, the fluorescence intensity showed no significant difference compared to that at 0 h (Fig. 2c). Fluorescence analysis of the lungs and femurs at various time points revealed distinct tissue distribution dynamics of 26SCS following oral administration. In the lungs, 26SCS rapidly accumulated, with peak fluorescence intensity observed at 6 h post administration, followed by a gradual decline and substantial clearance at 48 h (Fig. S2b, c). In the bone, maximal accumulation occurred at 12 h, after which the fluorescence intensity progressively decreased; at 24 h, it was markedly reduced, and became nearly undetectable at 48 h (Fig. 2d, e). Based on these findings, we chose a 24-hour interval for 26SCS gavage.

To determine the optimal therapeutic dosage, we performed a dose-gradient screening of 26SCS gavage treatments. Mice with pneumonia were treated with varying concentrations of 26SCS, and after 3 days, we measured IL-1β levels in their serum using enzyme-linked immunosorbent assay (ELISA). The results indicated that doses lower than 5 mg/kg led to a gradual increase in therapeutic efficacy as the concentration increased. However, doses exceeding 5 mg/kg showed subtle efficacy increase, so 5 mg/kg was selected as the optimal dose for gavage treatment (Fig. S2d). To assess the biocompatibility of the material, CCK-8 assays were performed, showing that SCS at concentrations of 1 μg/mL and 10 μg/mL exhibited no significant cytotoxicity toward macrophages (Fig. S2e). In addition, a safety evaluation was conducted by orally administering 26SCS at a dose of 10 mg/kg to healthy mice, followed by histological analysis of the heart, liver, spleen, lungs, and kidneys using hematoxylin-eosin (H&E) staining after 3 days (Fig. S2f). The results indicated that 26SCS gavage treatment did not induce any autoimmune reactions, and no evidence of significant toxicity was found, confirming the excellent biocompatibility and safety of 26SCS.^43^

DEX, as a classic anti-inflammation drug, was used here as a positive control. We treated mice with 26SCS, DEX, or PBS (negative control) via oral gavage 24 h after LPS stimulation (Fig. 2f). Micro-CT results revealed that 26SCS-treated mice showed more bone mass compared to the control and DEX groups on day 30, with superior trabecular architecture (Fig. 2g). Quantitative analysis showed that the 26SCS group exhibited higher bone volume fraction (BV/TV), trabecular number (Tb.N), and trabecular thickness (Tb.Th), as well as lower trabecular separation (Tb.Sp), with statistically significant differences (Fig. 2h). In contrast, the DEX-treated group showed more pronounced bone loss. Hematoxylin-eosin (H&E) staining of the femurs on days 3, 7, and 30 also revealed a trend of bone loss in ALI mice (Fig. 2i). Compared to the control and DEX groups, the 26SCS-treated group maintained a higher bone mass.

To further evaluate the effects of 26SCS on other skeletal sites, we additionally analyzed vertebral bone parameters in mice with acute lung injury following 26SCS treatment. μCT analysis revealed that 26SCS significantly ameliorated bone loss (Fig. S3a). Although there was no significant difference in Tb.N compared to the control group, marked improvements were observed in BV/TV, Tb.Sp, and Tb.Th. Notably, the Tb.Th value in the 26SCS group was comparable to that in the Mock group (Fig. S3b). These findings further support the systemic protective effect of 26SCS against pneumonia-induced bone loss.

### 26SCS inhibited ALI-triggered osteoclast activation

**Fig. 3:**
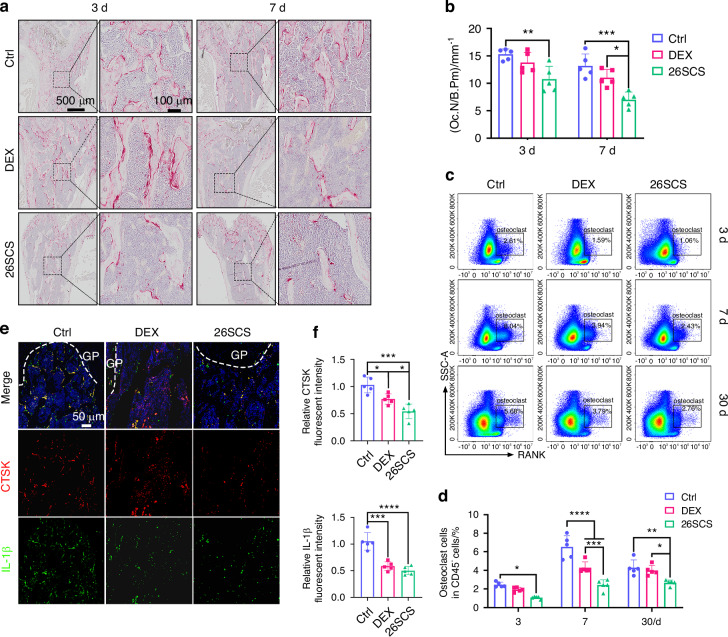
26SCS inhibits osteoclast activity induced by LPS-mediated ALI. **a** Representative TRAP-stained images of the distal femoral metaphysis on days 3 and 7 post-treatment with PBS (control group), DEX, or 26SCS. Insets display magnified regions. **b** Quantification of TRAP^+^ osteoclast numbers per bone perimeter (Oc.N/B.Pm) (*n* = 5). **c** Flow cytometry analysis of the percentage of RANK^+^ osteoclasts in the femurs on days 3, 7, and 30. **d** Quantitative analysis of the percentage of osteoclasts in CD45^+^ cells by flow cytometry (*n* = 5). **e** Representative immunofluorescence images of the distal femoral metaphysis on day 7, showing CTSK (red), IL-1β (green), and DAPI (blue). **f** Quantitative analysis of immunofluorescence intensities for CTSK and IL-1β in the distal femoral metaphysis (*n* = 5). Statistical analysis: Data are presented as mean ± standard deviation (SD). *P < 0.05, **P < 0.01, ***P < 0.005, ****P < 0.001

To characterize osteoclast activity in ALI mice, we performed tartrate-resistant acid phosphatase (TRAP) staining on femur sections at 3 and 7days post-treatment. The results revealed that the 26SCS group had significantly fewer TRAP^+^ osteoclasts compared to both the DEX and control groups (Fig. 3a, b). Flow cytometry analysis of osteoclasts in the femurs corroborated these findings, showing a lower proportion of osteoclasts in the 26SCS group, with a consistent downward trend (Figs. 3c and S4a). By day 7, while the DEX group exhibited a marked reduction in osteoclasts compared to the control group, it still had significantly more osteoclasts than the 26SCS-treated group (Fig. 3d). Overall, these results indicate that 26SCS effectively inhibits osteoclast activity induced by acute lung injury, with DEX treatment showing a weaker effect.

Given that the excessive osteoclast activity is primarily driven by the upregulation of inflammatory cytokines, we next used immunofluorescence staining to examine the expression of IL-1β and osteoclast marker CTSK in the femur (Fig. 3e). The results showed a clear co-localization of IL-1β and osteoclasts in the control group, indicating the association between inflammation and osteoclast activation. In contrast, 26SCS treatment reduced both IL-1β and CTSK expression, with statistically significant differences observed, suggesting that 26SCS treatment alleviates inflammation and consequently suppresses osteoclast activity (Fig. 3f). Taken together, 26SCS effectively mitigates ALI-induced bone loss, whereas DEX exacerbates bone resorption.

### 26SCS mitigates inflammation in both lung and bone

**Fig. 4:**
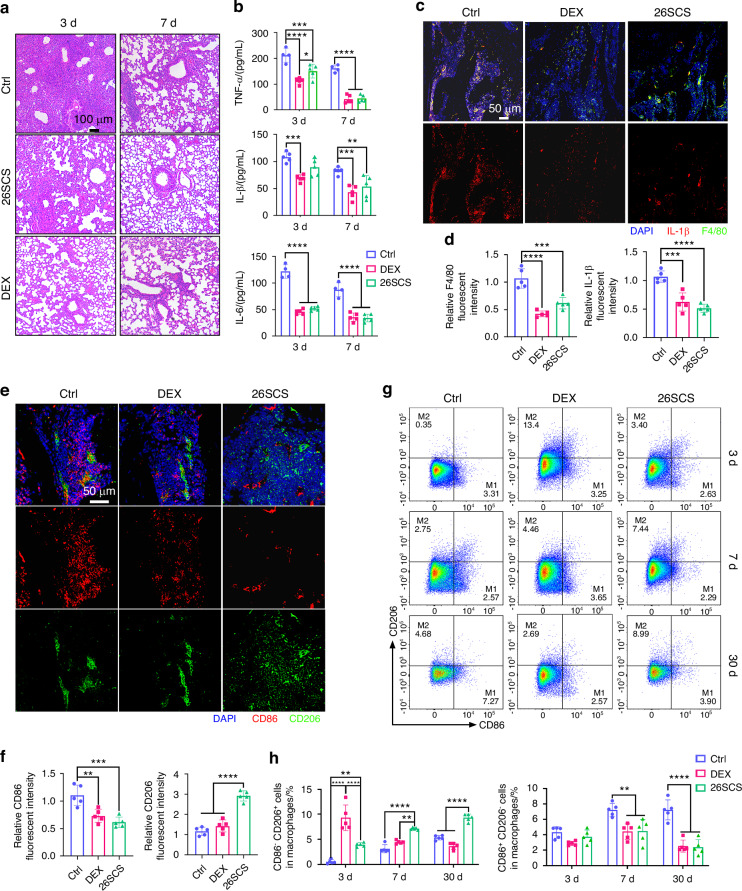
26SCS reduces inflammation caused by LPS-induced ALI. **a** Representative H&E staining images of lung tissues. **b** Serum levels of inflammatory cytokines (IL-6, TNF-α, and IL-1β) measured by ELISA on days 3 and 7 (*n* = 5). **c** Immunofluorescence staining of F4/80 (green), IL-1β (red), and DAPI (blue) in the femur on day 7. **d** Quantitative analysis of IL-1β and F4/80 fluorescence intensities in panel C (*n* = 5). **e** Immunofluorescence staining of CD86^+^ (red) and CD206^+^ (green) in bone marrow on day 7. **f** Quantitative analysis of fluorescence intensities of CD86 and CD206 in panel E (*n* = 5). **g** Flow cytometric analysis of CD86^+^ and CD206^+^ macrophages in bone marrow at different time points (days 3, 7, and 30). **h** Flow cytometric quantification of CD86⁺ and CD206⁺ macrophages in bone marrow (*n* = 5). Statistical analysis: Data are expressed as mean ± standard deviation (SD). *P < 0.05, **P < 0.01, ***P < 0.005, ****P < 0.001

As osteoclast activity is closely linked to inflammatory factors, we further explored the inflammatory response during the treatment process. Histological analysis using hematoxylin and eosin (H&E) staining of lung tissue in mice showed significant inflammatory cell infiltration, alveolar damage, and thickening of the alveolar walls in the control group. However, these inflammatory phenomena were markedly improved in both the 26SCS and DEX treatment groups (Fig. 4a). To further assess the levels of inflammatory cytokines, we conducted an enzyme-linked immunosorbent assay (ELISA) on bronchoalveolar lavage fluid (BALF) to measure the levels of IL-1β, TNF-α, and IL-6 (Fig. S5a). The results indicated a significant reduction in inflammatory cytokine levels in both the 26SCS and DEX treatment groups by day 3, with the DEX group showing a more pronounced anti-inflammatory effect. By day 7, the cytokine levels in both treatment groups were close to normal levels, with no significant differences between them. In contrast, although the control group exhibited a decrease in inflammatory cytokine levels, they remained elevated. To further investigate the regulatory effects of 26SCS on pulmonary inflammatory cells, flow cytometry was performed to assess macrophage polarization and neutrophil infiltration (Figs. S6 and S7). The results showed that 26SCS treatment led to a significant reduction in neutrophil infiltration at day 3, with levels approaching baseline at 7 days (Fig. S7a, c). In terms of macrophage polarization, the proportion of M1 macrophages in the 26SCS group was comparable to the Mock group on day 3, while M2 macrophages were significantly increased relative to the control group. At day 7, 26SCS treatment resulted in marked suppression of M1 polarization and enhancement of M2 polarization, indicating its potent immunoregulatory capacity (Fig. S7b, d).

We hypothesize that the elevated circulating inflammatory cytokines due to pneumonia play a crucial role in the activation of osteoclasts in the femur. Specifically, inflammatory factors from the lungs may escape into the bloodstream, enter the bone microenvironment through the circulatory system, and trigger a cascade of inflammatory responses that lead to osteoclast activation. To validate this hypothesis, we examined the levels of inflammatory cytokines in the serum using ELISA. The results showed that both 26SCS and DEX treatments significantly reducing serum cytokine levels induced by pneumonia (Fig. 4b). Additionally, blood profile analysis revealed that 26SCS significantly reduced white cell counts, neutrophil percentage, and monocyte percentage in circulation (Fig. S8a, b). We also investigated the inflammatory factor levels in the bone marrow. Immunofluorescence staining of F4/80 (a macrophage marker) and IL-1β in the femurs of mice on day 7 showed that both the 26SCS and DEX groups had significantly lower expression of IL-1β and macrophages compared to the control group (Fig. 4c, d). Interestingly, the 26SCS group seemed to exhibit lower IL-1β expression, while the DEX group appeared to show a more pronounced reduction in macrophage expression. Macrophages play essential roles in immune regulation and tissue repair, with plasticity that allows them to adopt either an M1 or M2 phenotype depending on the stimuli they encounter. M1 macrophages have pro-inflammatory functions and secrete cytokines like TNF-α, while M2 macrophages are involved in anti-inflammatory responses and tissue repair.^24,26^

To gain a better understanding of the bone microenvironment, we used immunofluorescence staining to analyze macrophage polarization in the femurs of mice at day 7 (Fig. 4e). The results revealed that 26SCS treatment promoted a higher M2 macrophage phenotype and a lower M1 macrophage phenotype (Fig. 4f). Additionally, we used flow cytometry to characterize the polarization trend of macrophages post-treatment (Fig. 4g and Fig. S5b). On day 3, there were no significant differences in the M1 macrophage proportion between the treatment and control groups. However, both 26SCS and DEX treatments significantly increased the proportion of M2 macrophages, with DEX showing a more pronounced effect, which corresponds to its stronger anti-inflammatory action. On days 7 and 30, both treatment groups showed significantly lower levels of M1 macrophages compared to the control group, with 26SCS promoting a more pronounced trend toward M2 polarization. DEX, however, did not exhibit this long-term effect, as the M2 macrophage levels in the DEX group were not significantly different from the control group after day 3 (Fig. 4h). Taken together, 26SCS promotes M1-to-M2 macrophage polarization, reduces inflammation, and fosters a favorable immune microenvironment for osteogenesis.

### 26SCS inhibited bone resorption by promoting M2 polarization of macrophages

**Fig. 5:**
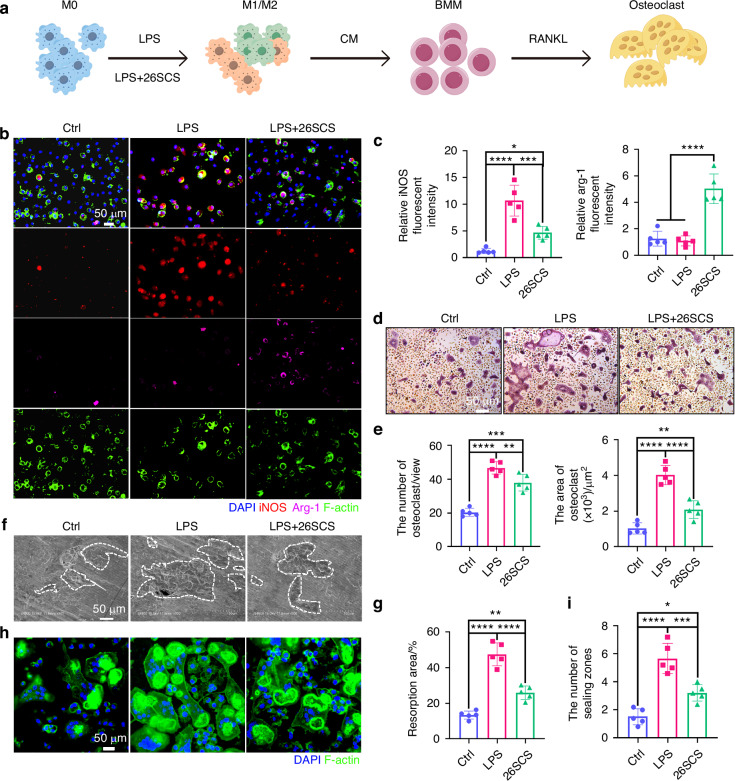
Mechanistic exploration of how 26SCS mitigates bone loss. **a** Schematic diagram of the experimental workflow: polarization of macrophages was induced by LPS with or without 26SCS treatment, followed by conditioned media collection for differentiation of monocytes into osteoclasts. **b** Representative immunofluorescence images of macrophages treated with LPS or LPS + 26SCS, showing DAPI (nuclei, blue), iNOS (M1 marker, red), Arg-1 (M2 marker, magenta), and F-actin (cytoskeleton, green). **c** Quantitative analysis of immunofluorescence intensities for iNOS and Arg-1 (*n* = 5). **d** TRAP staining images of osteoclasts cultured under indicated treatment conditions (Ctrl, LPS, LPS + 26SCS). **e** Quantitative analysis of osteoclast numbers and areas per field of view (*n* = 5). **f** Scanning electron microscopy (SEM) images of bone slices showing the resorption areas by osteoclasts. **g** Quantitative analysis of bone resorption area percentage (*n* = 5). **h** Immunofluorescence images of sealing zones in osteoclasts, marked by F-actin (green) and DAPI (blue). **i** Quantitative analysis of the number of sealing zones on bone slices (*n* = 5). Statistical analysis: Data are presented as mean ± standard deviation (SD). *P < 0.05, **P < 0.01, ***P < 0.005, ****P < 0.001

To further explore how 26SCS improves bone loss, we cultured bone marrow-derived macrophages under different conditions: LPS and LPS + 26SCS. A blank control group was also included. We used the conditioned media to culture bone marrow-derived monocytes to assess their osteoclast differentiation (Fig. 5a). We performed immunofluorescence staining on macrophages under different culture conditions to observe their phenotypic changes (Fig. 5b). The results showed that LPS significantly induced the polarization of M0 macrophages towards the pro-inflammatory M1 phenotype. However, when 26SCS was added, it notably increased the polarization of macrophages towards the anti-inflammatory M2 phenotype (Fig. 5c). To further investigate whether molecular weight influences biological function, LPS-stimulated macrophages were treated with sulfated chitosan oligosaccharide (SCOS), low-molecular-weight sulfated chitosan (LSCS), and sulfated chitosan (26SCS) (Fig. S9a). Immunofluorescence staining showed that all three effectively inhibited M1 polarization and promoted M2 polarization, with no statistically significant differences observed among them (Fig. S9b).

To further investigate the effect of different macrophage phenotypes on osteoclast differentiation, we assessed the differentiation capacity of monocytes cultured with conditioned media using TRAP staining (Fig. 5d). The results showed that, compared to the Mock group, both the LPS and 26SCS groups exhibited significantly increased osteoclast numbers. However, the 26SCS group exhibited smaller osteoclasts, which may contribute to reduced osteoclast-mediated bone resorption (Fig. 5e). To further evaluate this, we performed osteoclast induction cultures on bone slices and examined the degree of bone erosion using scanning electron microscopy (SEM) (Fig. 5f). The results indicated that, compared to the LPS group, the area of bone erosion by osteoclasts was significantly reduced following 26SCS treatment (Fig. 5g). Osteoclasts polarize to resorb bone through the formation of sealing zones (SZs) on the bone surface. Upon contact with the bone, osteoclasts extend their podosomes, which are adhesive, actin-rich structures, forming compact, highly dynamic podosome belts or rings to resorb bone. Immunofluorescence staining revealed that LPS not only induced the generation of more osteoclasts but also promoted the formation of more sealing zones. In contrast, the addition of 26SCS effectively inhibited both osteoclast formation and sealing zone formation, significantly reducing osteoclast bone resorption activity (Fig. 5h, i). Taken together, 26SCS promotes macrophage polarization towards the M2 anti-inflammatory phenotype, and thereby inhibits osteoclast activity, leading to reduced bone loss.

### 26SCS improves type H formation coupling with osteogenesis in ALI-induced inflammatory bone

**Fig. 6:**
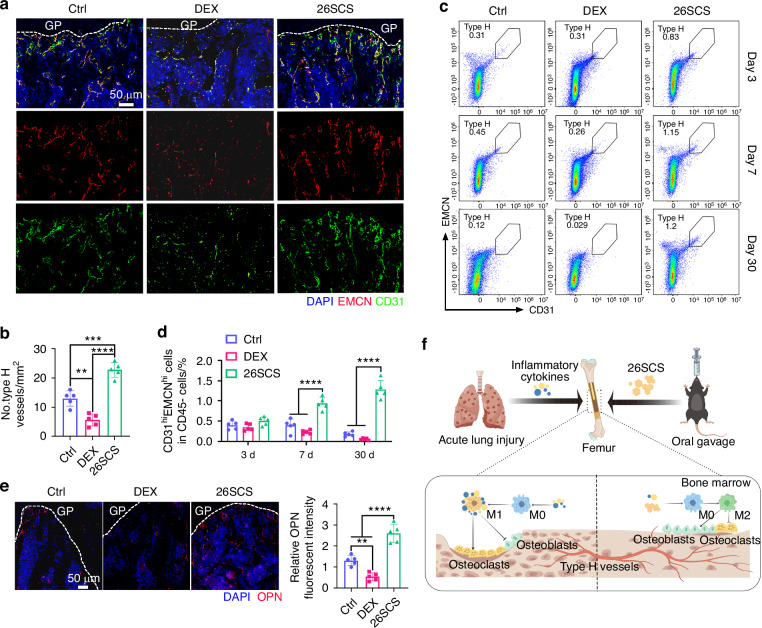
26SCS ameliorates type H recession in ALI-induced inflammatory bone. **a** Representative immunofluorescence images of the distal femoral metaphysis on day 7, showing CD31 (green) and EMCN (red). DAPI (blue) was used for nuclear staining. **b** Quantitative analysis of type H vessels in panel A (*n* = 5). **c** Flow cytometry analysis of type H blood vessels (CD31^hi^EMCN^hi^) in bone marrow at different time points (days 3, 7, and 30). **d** Quantitative analysis of the proportion of type H vascular cells in CD45⁻ cells using flow cytometry (*n* = 5). **e** Representative immunofluorescence images of the distal femoral metaphysis showing the bone formation marker OPN (red) and DAPI (blue) and quantitative analysis of the immunofluorescence intensity of OPN (*n* = 5). **f** Schematic illustration: 26SCS mitigates acute lung injury-induced bone loss by modulating macrophage functional states, thereby suppressing pro-inflammatory cytokine release and osteoclast overactivation, ultimately improving the inflammatory bone microenvironment. Statistical analysis: Data are presented as mean ± standard deviation (SD). *P < 0.05, **P < 0.01, ***P < 0.005, ****P < 0.001

Given the complexity and multifaceted nature of bone biology, as well as the unexplained phenomenon of more severe bone loss observed with DEX treatment, we conducted a more comprehensive exploration of the bone marrow microenvironment. Type H vessels, which were first identified in the skeletal system, are characterized by high expression of endothelial markers such as endothelial cell adhesion molecule (EMCN) and platelet-endothelial cell adhesion molecule (CD31).^44^ These vessels are known for their high oxygenation and permeability and have been shown to be closely related to bone homeostasis.^34^ We performed immunofluorescence staining to characterize Type H blood vessels in the femur (Fig. 6a). The results revealed that DEX treatment led to a significant downregulation of Type H blood vessels (Fig. 6b). To further investigate the trend of Type H vessel changes, we analyzed the data using flow cytometry (Figs. 6c and S10). On day 3, there were no significant differences between the groups in the proportion of CD31^hi^ EMCN^hi^ endothelial cells, with a slight increase in the 26SCS group. However, on days 7 and 30, the proportion of CD31^hi^ EMCN^hi^ endothelial cells in the 26SCS-treated group was significantly higher compared to both the control and DEX groups. Importantly, while the control group showed a gradual decline, the DEX group exhibited a sharp decrease, with only about 0.05% of Type H vessels remaining by day 30 (Fig. 6d). This suggests that DEX treatment severely disrupts type H blood vessels in the femur. To further investigate the mechanism by which 26SCS promotes Type H angiogenesis under inflammatory conditions, we collected conditioned medium from macrophages treated with 26SCS and applied it to Human Umbilical Vein Endothelial Cells (HUVECs) pre-stimulated with IL-1β to evaluate its effect on endothelial angiogenic capacity (Fig. S11a). The results showed that IL-1β stimulation significantly suppressed tube formation, as evidenced by reduced tube number and total tube area, indicating that the inflammatory microenvironment severely impairs endothelial angiogenic function. Notably, conditioned medium derived from 26SCS-treated macrophages effectively reversed this inhibition and markedly restored the tube-forming ability of HUVECs. These findings suggest that 26SCS indirectly enhances endothelial angiogenic function under inflammatory conditions by modulating macrophage activity (Fig. S11b).

Since type H blood vessels are closely associated with osteogenesis, we further analyzed osteoblast activity by immunofluorescence staining for osteoblast marker osteopontin (OPN). The results indicated a significant downregulation of osteoblasts following DEX treatment (Fig. 6e). Taken together, 26SCS promotes type H vessel formation and osteoblast activity, supporting bone formation, while DEX disrupts both, leading to increased bone loss. We investigated whether 26SCS exerts a direct effect on osteoblasts by performing alkaline phosphatase (ALP) staining on MC3T3 preosteoblasts treated with different concentrations of 26SCS (1 μg/mL and 10 μg/mL) (Fig. S12a). The results showed no significant difference between the 1 μg/mL group and the control, while the 10 μg/mL group exhibited increased ALP-positive staining compared to the control, but not significantly different from the 1 μg/mL group (Fig. S12b). These findings suggest that the direct pro-osteogenic effect of 26SCS on osteoblasts is limited, and its in vivo bone-promoting activity may primarily rely on indirect regulation via the vascular–bone interface or immune microenvironment.

## Disscusion

In this study, we identified inflammatory cytokines as key contributors to pneumonia-induced bone loss.^45^ Bone loss has been observed in conditions such as COVID-19, chronic fibrotic lung disease, asthma, and cystic fibrosis, yet the mechanisms underlying the bone-lung axis remain poorly understood.^7,8^ During pneumonia, inflammatory cytokines like IL-1β, IL-6, and TNF-α are released into the systemic circulation and infiltrate bone tissue, promoting macrophage polarization toward the pro-inflammatory M1 phenotype. This polarization triggers a cascade of inflammatory cytokine production, further amplifying inflammation. Even after pneumonia is resolved, sustained M1 macrophage polarization and cytokine production persist, likely driving prolonged osteoclast activation. Additionally, pneumonia-induced reduction in type H blood vessels impairs nutrient and oxygen supply, further disrupting bone metabolism (Fig. 6d). These findings emphasize that mitigating pneumonia-induced bone loss requires not only the inhibition of inflammatory cytokines in the lesion site but also the restoration of a healthy bone microenvironment.

Glucocorticoids like dexamethasone (DEX) are effective in controlling systemic inflammation, as seen in severe COVID-19 cases, but prolonged or high-dose use can lead to bone loss. Our study found that while DEX significantly reduced inflammatory cytokines in the lungs and serum, it caused severe bone loss and reduced Type H blood vessels, which are essential for osteoblast activity and bone formation. In contrast, 26SCS not only suppressed inflammation but also preserved the bone microenvironment, promoting Type H vessel formation and osteogenesis. Immunofluorescence staining revealed that 26SCS was more effective than DEX in promoting M2 macrophage polarization in bone tissue, contributing to a healthier bone microenvironment. These results highlight the superior therapeutic potential of 26SCS, which targets both inflammation and bone regeneration.

Macrophages are central regulators of bone health, with their phenotypes playing distinct roles in inflammation and repair.^25^ M1 macrophages, activated during acute inflammation, are crucial for pathogen clearance but can perpetuate tissue damage and chronic inflammation if overactivated. Conversely, M2 macrophages resolve inflammation and promote tissue repair by secreting anti-inflammatory cytokines like IL-10 and TGF-β.^24,26^ Our study demonstrates that 26SCS effectively promotes M2 macrophage polarization, reduces pro-inflammatory cytokines, and mitigates osteoclast overactivation, creating a more favorable immune microenvironment for bone health. Compared to DEX, which suppresses systemic inflammation but disrupts the bone microenvironment, 26SCS offers a more comprehensive therapeutic approach. It not only dampens systemic inflammation but also enhances M2-mediated VEGF secretion, supporting type H blood vessel formation that couples angiogenesis with osteogenesis.^35^ These findings highlight the dual action of 26SCS in immune modulation and bone regeneration, providing a significant advantage over traditional anti-inflammatory treatments. Further investigation into the molecular pathways driving these effects could expand its therapeutic applications for inflammation-induced bone disorders.

This study demonstrates that orally administered 26SCS mitigates pneumonia-induced bone loss by promoting M2 macrophage polarization, reducing inflammatory cytokines, suppressing osteoclast activity, and enhancing type H blood vessel formation. These actions create a favorable immune and vascular microenvironment that supports bone formation. Future research will explore the mechanisms by which 26SCS influences macrophages, including its systemic effects and potential direct action on bone marrow macrophages.

## Materials and methods

### The acute lung injury animal model

C57 male mice, aged 6-8 weeks, were purchased from Shanghai Jiesijie Experimental Animal Co., Ltd. The animal model construction was supervised by the Animal Research Committee of East China University of Science and Technology. Mice were anesthetized via intraperitoneal injection of sodium pentobarbital (3 mg/kg), with their upper incisors suspended on a support frame. The tongue was gently extended using forceps, and the glottis was identified using a transmitted light. A catheter was passed through the glottis, the metal needle was removed, and lipopolysaccharide (LPS, 10 mg/kg) was injected via a micro-syringe. The mice were suspended for 5-10 seconds and then placed on a constant temperature heating plate until they recovered, after which they were transferred to cages. Subsequently, the mice were orally administered PBS, 26SCS (5 mg/kg), or DEX (5 mg/kg) daily for two weeks. Samples were collected at 3, 7, and 30 days post-treatment.

### The synthesis of 2-N,6-O-sulfated chitosan

The sulfonation reagent was first prepared. Briefly, 5 mL of chlorosulfonic acid was slowly added dropwise to 5 mL of DMF in a three-neck flask under an ice bath at 0-4 °C, and the mixture was stirred until the reaction was complete, then allowed to cool to room temperature. Chitosan was then weighed and transferred to another three-neck flask, where formamide and formic acid were added. The previously prepared sulfonation reagent was slowly added, and the reaction was stirred thoroughly at 50 °C. After completion, anhydrous ethanol was added to precipitate the product, which was then vacuum filtered. The solid was washed multiple times, dissolved in a small amount of ultrapure water, and centrifuged to discard the insoluble material. This process was repeated several times, and the supernatant was transferred to a dialysis bag for three days of dialysis. The pH was adjusted to 7-8, and the product was freeze-dried. The resulting product was characterized using Fourier transform infrared (FTIR) spectroscopy, nuclear magnetic resonance (NMR) hydrogen spectroscopy, and elemental analysis to verify the synthesis and degree of sulfation.

To obtain sulfated chitosan fractions with different molecular weights, a stepwise dialysis method was employed based on molecular weight cut-off (MWCO). Briefly, 26SCS was dissolved in deionized water and sequentially dialyzed using dialysis membranes with MWCOs of 25 kD, 8 kD, 5 kD and 1 kD. The fraction retained by the 25 kD membrane was collected as high molecular weight 26SCS. The fraction that passed through the 25 kD membrane but was retained by the 8 kD membrane was designated as LSCS. Similarly, the fraction between 1 kD and 3 kD was defined as SCOS. All dialyzed samples were lyophilized and stored at −20 °C until use.

### Micro-CT analysis

According to the previously described standard guidelines, after 30 days of treatment, the femora and L4 vertebrae of the mice were harvested and fixed in 4% paraformaldehyde. Micro-computed tomography (μCT) scanning was performed at the Shanghai Synchrotron Radiation Facility using a 26 keV energy beam. The pixel size was set to 5.5 μm, and the images were recorded for tomography. AVIZO software was used for 3D model visualization. The region of interest (ROI) included the growth plate from the distal femoral diaphysis. The trabecular bone parameters were measured from the μCT data, including bone volume/total volume (BV/TV), trabecular thickness (Tb.Th), trabecular number (Tb.N), and trabecular separation (Tb.Sp).

### Histological analysis

For histological staining of paraffin sections, the bone specimens were fixed in 4% PFA for 48 h, followed by decalcification in 12.5% ethylenediaminetetraacetic acid (EDTA, Sigma-Aldrich) for 4 weeks. For paraffin sections, the samples were processed, embedded in paraffin, and cut into 5 μm thick sections using a rotary microtome. Hematoxylin and eosin (H&E) staining, TRAP staining, and ALP staining were performed on selected sections from each sample according to the manufacturer’s instructions. Microscopic imaging was conducted using an APEXVIEW APX100 imaging system.

### Immunohistochemistry (IHC) analysis

For immunohistochemistry staining, paraffin-embedded tissue samples were processed according to the manufacturer’s instructions for heat fixation, deparaffinization, rehydration, antigen retrieval, and subsequent steps. First, tissue sections were heat-fixed at 60 °C for 2 h, deparaffinized in xylene for 30 minutes, and then rehydrated in 100%, 75%, and 50% ethanol for 10 minutes each, followed by washing with distilled water for 5 minutes. Antigen retrieval was performed in a 10 × 10^-3^ mol/L citrate buffer solution under high pressure for 2 minutes. The sections were incubated with 3% hydrogen peroxide in the dark for 10 minutes, blocked with 3% goat serum for 1 h, and then incubated overnight at 4 °C with primary antibodies against IL-1β, TNF-α, and IL-6. Afterward, sections were washed three times with phosphate-buffered saline containing 0.05% Tween-20 (PBST). A 50 μL solution of HRP-conjugated goat anti-mouse/anti-rabbit IgG was added to the sections and incubated for 30 minutes, followed by three washes with PBST. Subsequently, 50 μL of 3,3’-diaminobenzidine (DAB) reagent was added for color development, and the sections were counterstained with hematoxylin for 30 seconds. Finally, the sections were dehydrated by sequential immersion in 50%, 75%, and 100% ethanol and xylene for 10 minutes each. Images of the samples were captured using a Leica fluorescence optical microscope.

### Immunofluorescence analysis

For frozen section immunostaining, mouse femora were fixed in cold 4% paraformaldehyde for 24 h, decalcified in 15% ethylenediaminetetraacetic acid (EDTA) for 3 days, with daily solution changes. The specimens were then immersed in 20% sucrose containing 2% polyvinylpyrrolidone (PVP) for dehydration at 4 °C for 12 h. Subsequently, they were embedded in OCT compound (Sakura, USA) and cut into 7 μm thick sections using a cryostat. Standard immunostaining protocols were followed. Briefly, after blocking with 10% goat serum, the sections were incubated overnight at 4 °C with primary antibodies against F4/80, IL-1β, CTSK, CD86, CD206, EMCN, CD31, OPN, iNOS, Arg-1, and phalloidin. Alexa-Fluor 488-conjugated, Alexa-Fluor 555-conjugated, and Alexa-Fluor 647-conjugated secondary antibodies were used for immunofluorescence staining, and the cell nuclei were counterstained with DAPI. Immunofluorescence images were captured using the LSM 780 confocal microscope.

### Flow cytometry

Cell recruitment and phenotyping in femora were assessed by flow cytometry. Briefly, mouse femora were placed in 1 mL of PBS and ground, followed by centrifugation at 300 *g* for 5 minutes. The supernatant was collected and stored at -80 °C for further analysis. The tissue pellet was lysed with 1 mL of red blood cell lysis buffer for 2 minutes, and after centrifugation at 300 *g* for 5 minutes, 1 mL of buffer was added to obtain a cell suspension. The cell suspension was then filtered through a 40 μm mesh and centrifuged again, with 100 μL of buffer added to obtain a single-cell suspension. For macrophage staining, the cell suspension was stained with Live/Dead, FITC-conjugated CD45, Alexa Fluor 700-conjugated CD11b, APC-conjugated F4/80, PE-conjugated CD86, and BV421-conjugated CD206 for 45 minutes at 4 °C. For the vascular group, cells were incubated with EMCN primary antibody at 4 °C for 30 minutes, followed by the addition of AF647-conjugated secondary antibody. Additionally, Live/Dead, FITC-conjugated CD45, and PE/CY7-conjugated CD31 were used for 45 minutes incubation. For osteoclast staining, cells were incubated with RANK primary antibody for 30 minutes, followed by the addition of AF555-conjugated secondary antibody, along with Live/Dead and FITC-conjugated CD45 for 45 minutes.

Mouse lung tissues were excised and finely minced, then incubated at 37 °C for 35 minutes in a digestion solution containing collagenase D and DNase I. The digestion was terminated, and the cell suspension was filtered through a 70 μm cell strainer, followed by centrifugation at 300 *g* for 15 minutes to collect single cells. Red blood cells were lysed by adding 2 mL of lysis buffer for 2 minutes, then centrifuged at 300 *g* for 5 minutes. The cell pellet was resuspended in 1 mL of buffer to obtain a single-cell suspension. For macrophage and neutrophil staining, the cell suspension was stained with Live/Dead, FITC-conjugated CD45, Alexa Fluor 700-conjugated CD11b, APC-conjugated F4/80, PE-conjugated CD86, BV421-conjugated CD206 and PE/Cy7-conjugated Ly6G for 45 minutes at 4 °C. The cell suspensions were then analyzed on a Cytoflex LX (Beckman Coulter, USA) flow cytometer, and data were analyzed using FlowJo software (Tree Star).

### ELISA analysis

Bronchoalveolar lavage fluid (BALF) and serum were collected from mice at 3 or 7 days. Additionally, cell culture medium was collected for cytokine/chemokine analysis. Serum levels of IL-1β, TNF-α, and IL-6 were measured using specific ELISA kits (according to the manufacturer’s instructions).

### Femoral enrichment of 26SCS

To evaluate whether the polysaccharide accumulates in the lungs and bones, we orally administered fluorescence-labeled 26SCS (5 mg/kg) to mice. At 6 h, 12 h, 24 h, and 48 h post-administration, femurs were collected and analyzed using the IVIS Spectrum Fluorescence Imaging System (PerkinElmer, USA) to monitor changes in the fluorescence signal in the femurs.

### Blood fluorescence intensity measurement

Fluorescent 26SCS (5 mg/kg) was administered via gavage. After 6 h, 50 μL of blood was collected from the orbital sinus, mixed with 50 μL of PBS containing 0.2 × 10⁻³ mol/L EDTA.2 K (anticoagulant). The absorbance of peripheral blood was measured using a microplate reader.

### Cell culture

Macrophage Isolation: Briefly, after euthanizing the mice, their limbs were sterilized with 75% ethanol, and the femur and tibia were isolated and placed in a culture dish containing buffer (2% serum + PBS). The ends of the femur and tibia were trimmed, and the bones were flushed with 1 mL syringe and 5 mL medium (10% serum +1% antibiotics + DMEM) at both ends. The tissue fragments were separated by filtration. The suspension was centrifuged at 300 *g* for 5 minutes, and the supernatant was discarded. The cells were resuspended in medium and cultured in a large culture dish for 24 h. Non-adherent cells (mononuclear cells) were collected by centrifugation at 300 *g* for 5 minutes and the supernatant was discarded. The cells were cultured overnight in medium containing 30 ng/mL M-CSF to obtain macrophages.

Macrophage cytotoxicity assay: Macrophages were seeded into 96-well plates and cultured overnight to allow cell attachment. Cells were then treated with 1 μg/mL or 10 μg/mL SCS for 1, 3, or 7 days. At each time point, CCK-8 reagent was added according to the manufacturer’s instructions, followed by incubation at 37 °C for 2 h. The absorbance at 450 nm was measured using a microplate reader to assess cell viability.

Macrophage Immunofluorescence Staining: Briefly, Cells were seeded into sterile glass-bottom 96-well plates and treated with LPS, or LPS in combination with 26SCS, LSCS, or SCOS. After reaching appropriate confluency, the medium was removed and cells were washed with PBS, followed by fixation with 4% paraformaldehyde for 10 minutes and permeabilization with 0.1% Triton X-100 for 10 minutes. After blocking with 10% goat serum, cells were incubated overnight at 4 °C with primary antibodies against iNOS and Arg-1. Alexa Fluor 488-conjugated and Alexa Fluor 647-conjugated secondary antibodies were used for immunofluorescence staining, and nuclei were counterstained with DAPI. Images were acquired using a fluorescence microscope.

Osteoclast TRAP Staining: Briefly, cells were cultured for 1 day in LPS (1 μg/mL), LPS + 26SCS (800 ng/mL), or Ctrl medium. After 2 days of culture in conditioned medium (2% serum), the medium was removed, and the cells were centrifuged at 1 000 r/min for 5 minutes to remove impurities. The cell pellet was stored at -80 °C for further use. After supplementation of the conditioned medium to 10% serum, 100 ng/mL RANKL and 50 ng/mL M-CSF were added to 96-well plates to induce osteoclastogenesis from mononuclear cells. The medium was replaced the next day, and after 7 days of culture, the cells were subjected to TRAP staining to observe osteoclast formation.

Osteoclast Immunofluorescence Staining: Similarly, using the aforementioned conditioned medium, mononuclear cells were cultured on bone slices. After 7 days, the bone slices containing cells were fixed in paraformaldehyde. The bone slices were then treated with FITC-labeled cytochalasin D and DAPI for immunofluorescence staining, followed by confocal microscopy imaging. Additionally, surface cells were detached using cell dissociation enzyme, and the bone slices were subjected to graded dehydration. Scanning electron microscopy (SEM) was then used to observe the bone slices.

MC3T3 ALP Staining: Briefly, MC3T3-E1 cells were seeded into 6-well plates at a density of 50 000 cells/cm². After reaching approximately 90% confluence, the medium was replaced with osteogenic induction medium. The treatment groups received either 1 μg/mL or 10 μg/mL of the test compound, while the control group received induction medium only. The medium was changed every 3 days. After 7 days of induction, ALP staining was performed. Cells were washed with PBS and fixed with 4% paraformaldehyde for 15 minutes at room temperature, followed by three PBS washes (5 minutes each). BCIP/NBT staining solution was then added, and cells were incubated at 37 °C in the dark for 60 minutes. The reaction was stopped by rinsing with distilled water, and ALP activity was visualized as purple precipitates under a light microscope.

### Quantification and statistical analysis

All data analysis was performed using Prism software (version 7, GraphPad, USA) and is described accordingly. Results are presented as mean ± standard deviation (SD). The exact sample size (*n*) for each experimental group is clearly shown in the dot plot and indicated in the figure legend. Unpaired two-tailed Student’s t-test was used for comparisons between two groups. To compare multiple experimental groups, one-way or two-way analysis of variance (ANOVA) was performed where indicated. P-values < 0.05 were considered statistically significant: *P < 0.05, **P < 0.01, ***P < 0.005, and ****P < 0.001.

## Supplementary information

Supplementary information

Figure S1

Figure S2

Figure S3

Figure S4

Figure S5

Figure S6

Figure S7

Figure S8

Figure S9

Figure S10

Figure S11

Figure S12
