# Supplementary material for: Sulfated chitosan mitigates acute lung injury induced bone loss via immunoregulation
Source: Bone Res. 2026 Feb 5;14:18. doi: 10.1038/s41413-025-00475-4 (PMC12877068; doi:10.1038/s41413-025-00475-4)
Supplement: Supplementary file 1 — Supplementary information [file 41413_2025_475_MOESM1_ESM.docx]

**SUPPLEMENTARY INFORMATION**

| REAGENT OR RESOURCE | SOURCE | IDENTIFIER |
| --- | --- | --- |
| Antibodies | | |
| Anti-mouse CD45 | Biolegend | Cat: 103108; RRID: AB_312972 |
| Anti-mouse CD11b | Biolegend | Cat: 101222; RRID: AB_493705 |
| Anti-mouse F4/80 | Biolegend | Cat: 123116; RRID: AB_893481 |
| Anti-mouse CD86 | Biolegend | Cat: 105008; RRID: AB_313150 |
| Anti-mouse CD206 | Biolegend | Cat: 141717; RRID: AB_2562232 |
| Anti-mouse CD31 | Biolegend | Cat: 102523; RRID: AB_2572181 |
| Anti-mouse EMCN | proteintech | Cat: 67854-1-Ig |
| Anti-mouse RANK | [Santa Cruz](https://www.scbt.com/zh/home) | Cat: sc-52951 |
| LIVE/DEAD^TM^ | Invitrogen | Cat: L10119 |
| Anti-mouse F4/80 | proteintech | Cat: 28463-1-AP |
| Anti-mouse IL-1β | Servicebio | Cat: GB12115-100 |
| Anti-mouse CTSK | proteintech | Cat: 11239-1-AP |
| Anti-mouse CD86 | abcam | Cat: ab119857 |
| Anti-mouse CD31 | proteintech | Cat: 80530-1-RR |
| Anti-mouse CD206 | abcam | Cat: ab64693 |
| Anti-mouse iNOS | proteintech | Cat: 18985-1-AP |
| Anti-mouse Arg-1 | proteintech | Cat: 66129-1-Ig |
| Anti-mouse OPN | proteintech | Cat: 30200-1-AP |
| Biological samples | | |
| Murine organs | Mice in this study | N/A |
| Murine femurs | Mice in this study | N/A |
| Murine serum | Mice in this study | N/A |
| Murine macrophages | Mice in this study | N/A |
| Murine monocytes | Mice in this study | N/A |
| Chemicals, peptides, and recombinant proteins | | |
| Chitosan (Mw: ~30 × 10^4^ Da) | Shenzhen Zhongfayuan | 9012-76-4 |
| OCT compound | Sakura | 4583 |
| 4% paraformaldehyde buffer | Servicebio | G1101-500ML |
| staining buffer | BioLegend | 420201 |
| DEX | Maokangbio | MX3251 |
| LPS | Sigma Aldrich | 297-473-0 |
| DMEM medium | Gibco |  |
| EDTA | Servicebio | G1105-500ML |
| Critical commercial assays | | |
| Mouse IL-1 beta ELISA Kit | proteintech | Cat: KE10003 |
| Mouse TNF-alpha ELISA Kit | proteintech | Cat: KE10002 |
| Mouse IL-6 ELISA Kit | proteintech | Cat: KE10007 |
| Cell Counting Kit-8 | Servicebio | Cat：G4103 |
| Software and algorithms | | |
| FlowJo | Tree Star | [Home \| FlowJo, LLC](https://www.flowjo.com/) |
| Microsoft office | Microsoft | https://www.microsoft.com/ |
| GraphPad Prism 9.0 | GraphPad | http://www.graphpad-prism.cn/ |
| Adobe Photoshop | Adobe | https://www.adobe.com/cn/ products/photoshop.html |
| Adobe Illustrator | Adobe | https://www.adobe.com/cn/ products/illustrator.html |
| ChemDraw | Revvity | https://www.chemdraw.com. |
| ImageJ | National Institutes of Health | https://imagej.net |

|  |
| --- |

Figure. S1. Effects of LPS-induced acute lung injury on bone inflammation and histology. **a** Representative immunohistochemical staining images of femurs showing IL-1β, TNF-α, and IL-6 expression under mock and LPS-treated conditions. **b** Quantification of IL-1β, TNF-α, and IL-6 positive areas in femoral sections (n=5). **c** Representative H&E staining images of femoral sections showing overall bone structure and inflammatory changes. Insets represent higher magnification of boxed areas. Statistical analysis: Data are presented as mean ± standard deviation (SD). *P < 0.05, **P < 0.01, ***P < 0.005, **** < 0.001.

|  |
| --- |

Figure. S2. Biocompatibility, sulfation degree, and dosage optimization of 26SCS. **a** Sulfation degree analysis of 26SCS. **b** Ex vivo fluorescence imaging of lung tissue harvested at different time points (6, 12, 24, and 48 hours) after oral administration of fluorescence-labeled 26SCS. **c** Time course of fluorescence intensity in lung tissue after oral administration (n=3). **d** Effect of different doses of 26SCS on cytokine concentration (n=3). **e** CCK-8 assay of macrophage viability after treatment with 26SCS (1 and 10 μg/mL) for 1, 3, and 7 days (n=5). **f** Representative H&E staining images of the heart, liver, spleen, lung, and kidney tissues from the Ctrl and 26SCS groups. Statistical analysis: Data are presented as mean ± standard deviation (SD).

|  |
| --- |

Figure. S3. 26SCS mitigates bone loss in vertebrae. **a** Representative μCT images of L4 vertebrae on day 30. **b** Quantitative analysis of bone parameters based on μCT, including bone volume fraction (BV/TV), trabecular separation (Tb.Sp), trabecular thickness (Tb.Th), and trabecular number (Tb.N) (n=5). Statistical analysis: Data are presented as mean ± standard deviation (SD). *P < 0.05, **P < 0.01, ***P < 0.005, **** < 0.001.

|  |
| --- |

Figure. S4. **a** The logic of flow cytometry gating for osteoclast.

|  |
| --- |

Figure. S5. **a** BALF levels of inflammatory cytokines (TNF-α, IL-1β, IL-6) measured by ELISA on days 3 and 7 (n=5). **b** The logic of flow cytometry gating for macrophage polarization in the femurs. Statistical analysis: Data are presented as mean ± standard deviation (SD). *P < 0.05, **P < 0.01, ***P < 0.005, **** < 0.001.

|  |
| --- |

Figure. S6. The logic of flow cytometry gating for macrophage polarization and neutrophil infiltration in the lungs.

|  |
| --- |

Figure. S7. Flow cytometric analysis of macrophage polarization and neutrophil infiltration in the lungs. **a** Flow cytometric analysis and quantification of M1 and M2 macrophages in lung tissue on day 3 (n=5). **b** Flow cytometric analysis and quantification of neutrophils in lung tissue on day 3 (n=5). **c** Flow cytometric analysis and quantification of M1 and M2 macrophages in lung tissue on day 7 (n=5). **d** Flow cytometric analysis and quantification of neutrophils in lung tissue on day 7 (n=5). Statistical analysis: Data are presented as mean ± standard deviation (SD). *P < 0.05, **P < 0.01, ***P < 0.005, **** < 0.001.

|  |
| --- |

Figure. S8. Peripheral blood routine analysis. **a** Quantification of peripheral blood immune cell populations on day 3 (n=5). **b** Quantification of peripheral blood immune cell populations on day 3 (n=5). Statistical analysis: Data are presented as mean ± standard deviation (SD). *P < 0.05, **P < 0.01, ***P < 0.005, **** < 0.001.

| ­ |
| --- |

Figure. S9. Effect of SCS with different molecular weights on macrophage polarization. **a** Representative immunofluorescence images of macrophages treated with different molecular weight of SCS, showing iNOS (green), Arg-1 (red), and DAPI (blue). **b** Quantitative analysis of immunofluorescence intensities for iNOS and Arg-1 (n=5). Statistical analysis: Data are presented as mean ± standard deviation (SD). *P < 0.05, **P < 0.01, ***P < 0.005, **** < 0.001.

|  |
| --- |

Figure. S10. **a** ­The logic of flow cytometry gating for Type H endothelial cells.

|  |
| --- |

Figure. S11. 26SCS promotes tube formation of HUVECs in vitro. **a** Representative images of tube formation by human umbilical vein endothelial cells (HUVECs) under different treatments, observed after 6 hours. Scale bar: 200 μm. **b** Quantification of the number of loops and the relative area of tubes formed (n=5). Statistical analysis: Data are presented as mean ± standard deviation (SD). *P < 0.05, **P < 0.01, ***P < 0.005, **** < 0.001.

|  |
| --- |

Figure. S12. Effects of 26SCS on ALP activity in MC3T3 cells. **a** Representative ALP staining images after 7-day treatment with different concentrations (1 μg/mL and 10 μg/mL) of SCS. **b** Quantification of ALP-positive area percentage (n=5). Statistical analysis: Data are presented as mean ± standard deviation (SD). *P < 0.05, **P < 0.01, ***P < 0.005, **** < 0.001.
