# Supplementary figures and images for: Sulfated chitosan mitigates acute lung injury induced bone loss via immunoregulation
Source: Bone Res. 2026 Feb 5;14:18. doi: 10.1038/s41413-025-00475-4 (PMC12877068; doi:10.1038/s41413-025-00475-4)

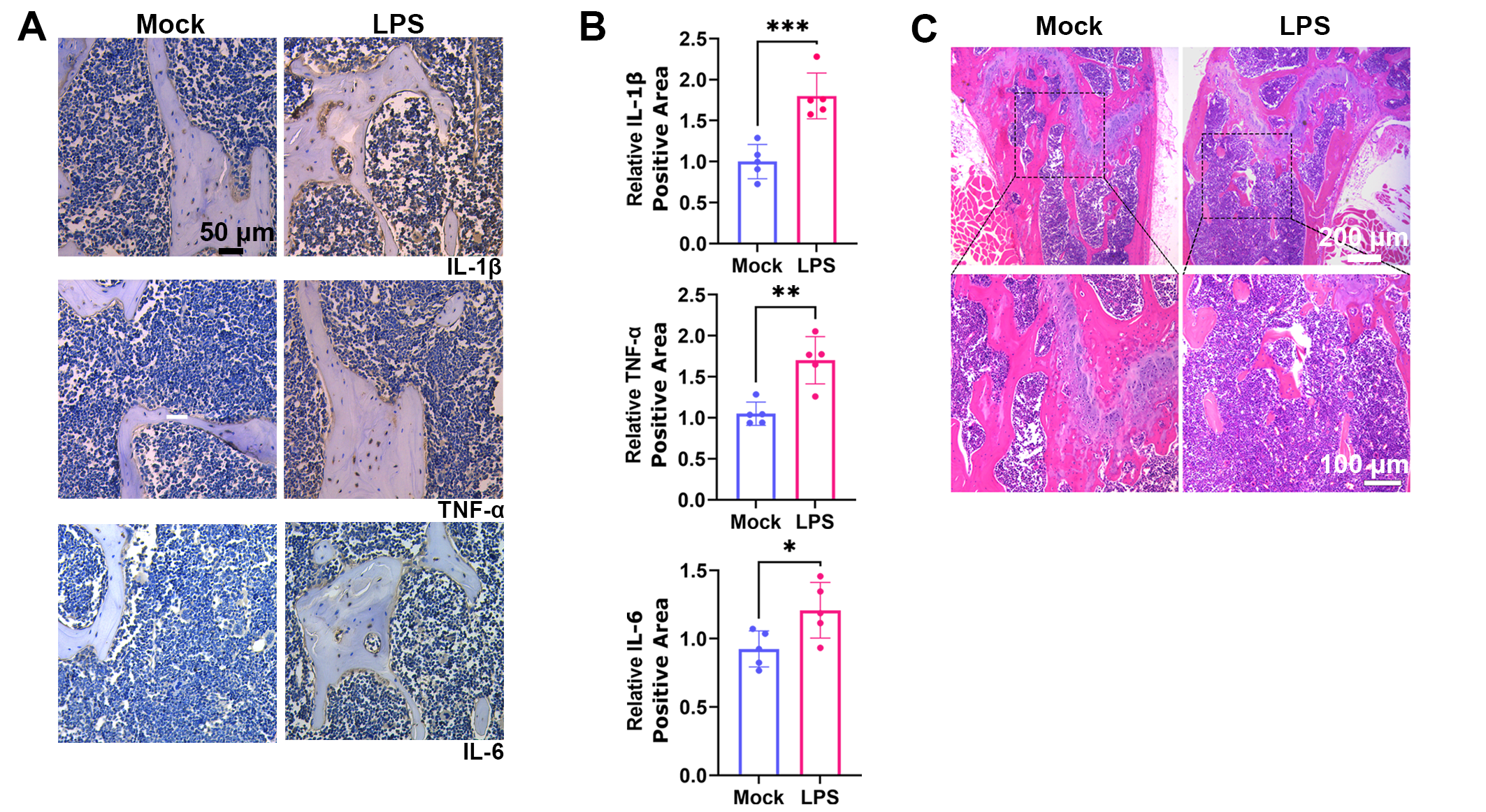

Supplement: Supplementary file 2 — Figure S1 [file 41413_2025_475_MOESM2_ESM.tif]

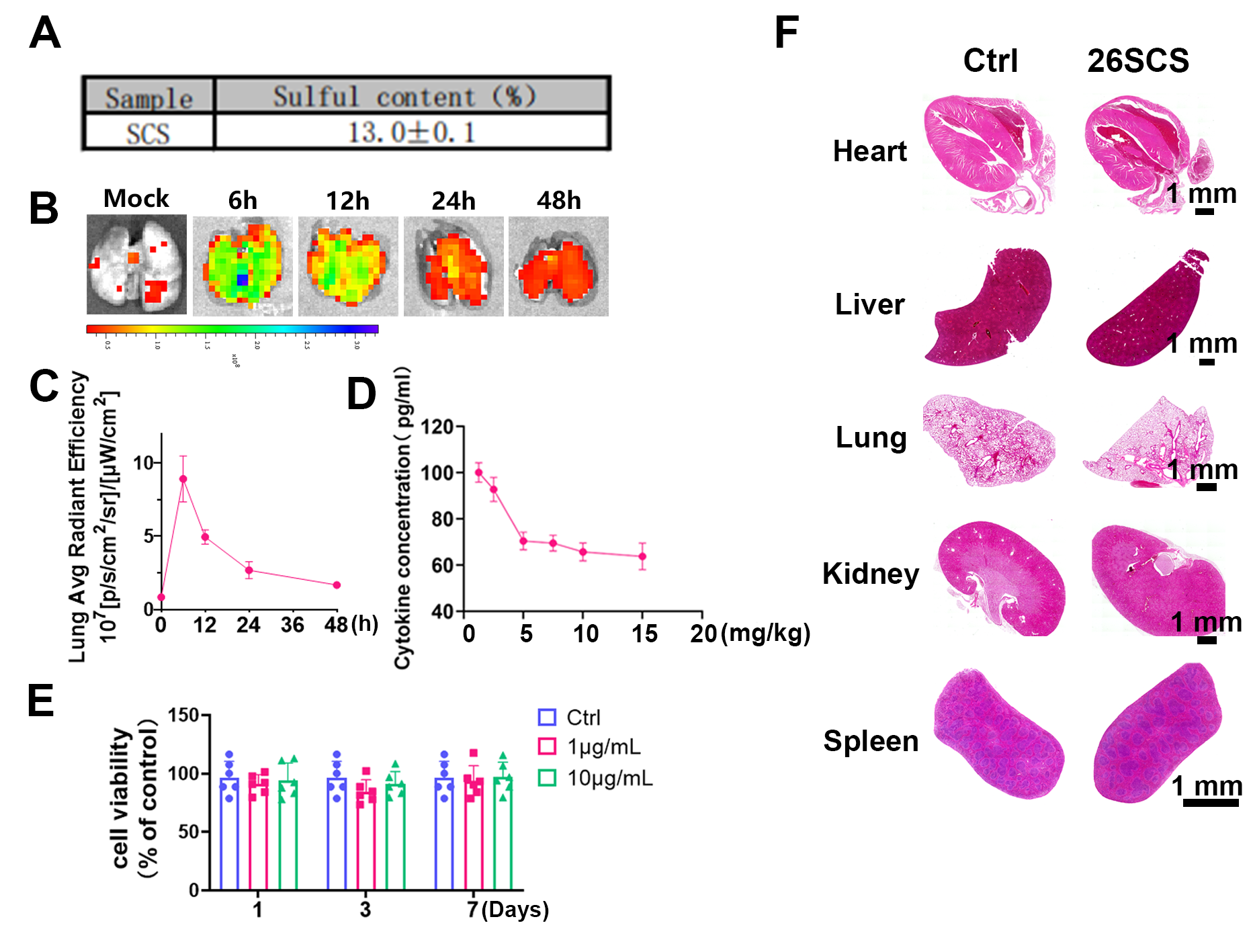

Supplement: Supplementary file 3 — Figure S2 [file 41413_2025_475_MOESM3_ESM.tif]

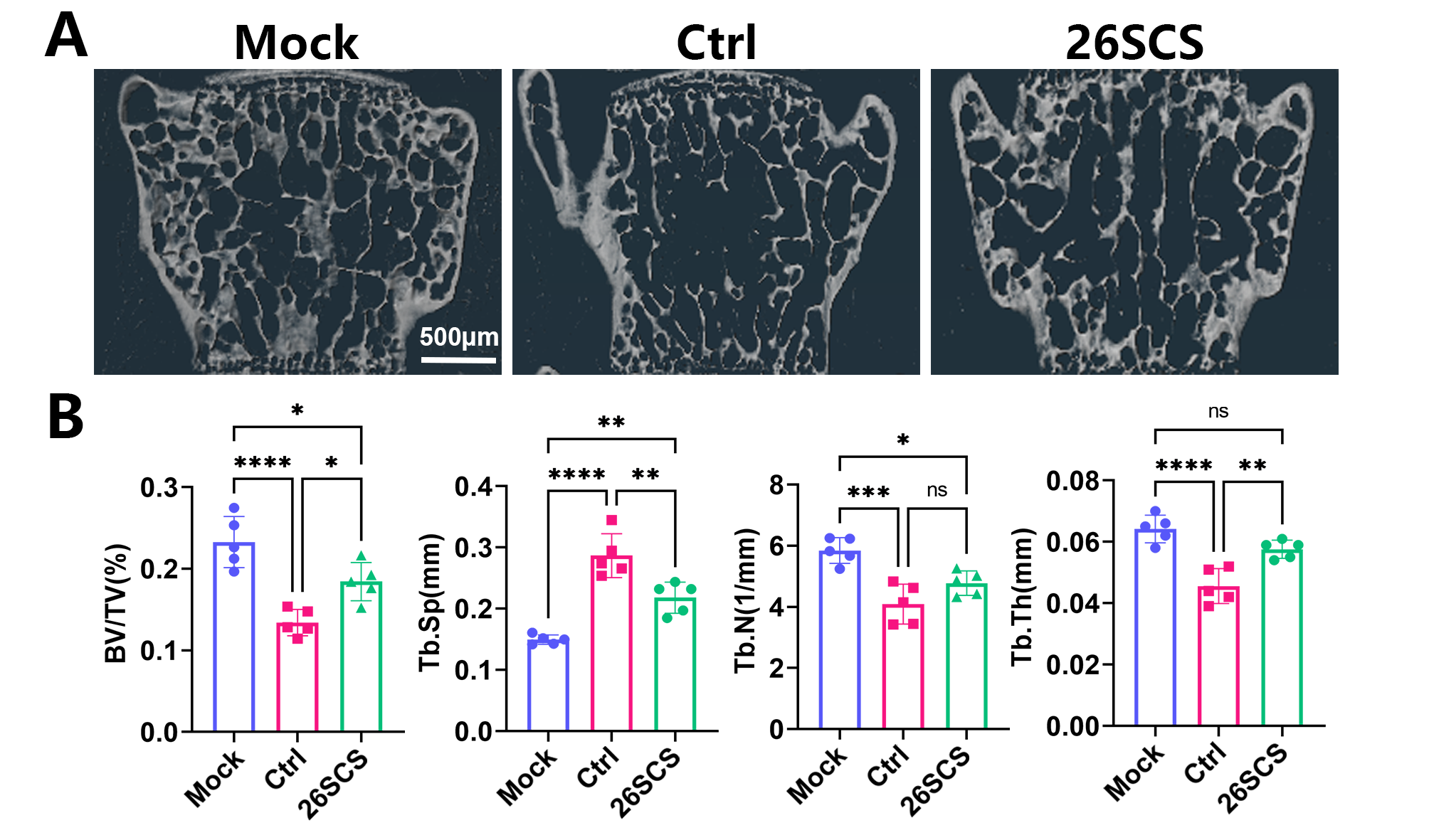

Supplement: Supplementary file 4 — Figure S3 [file 41413_2025_475_MOESM4_ESM.tif]

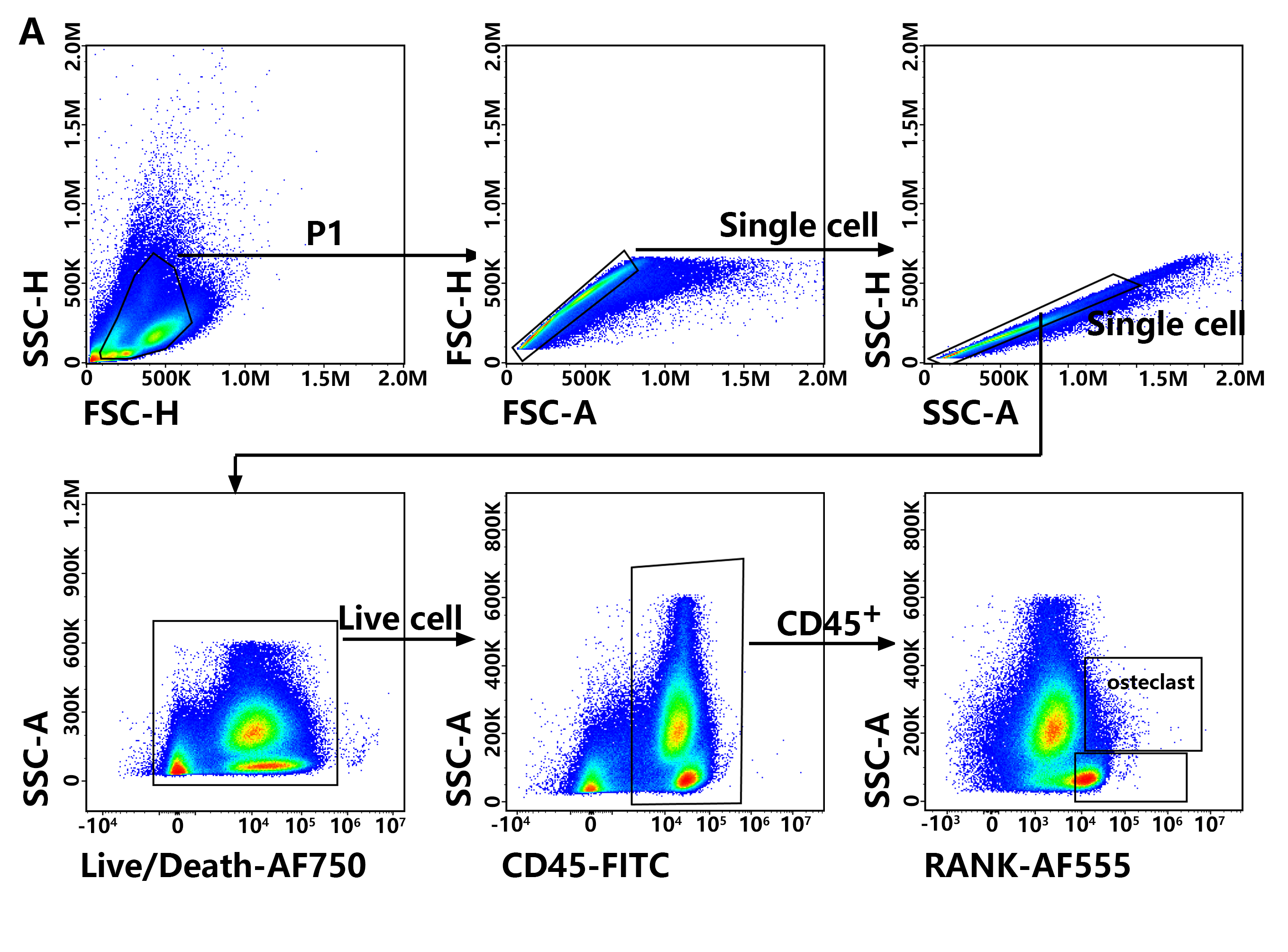

Supplement: Supplementary file 5 — Figure S4 [file 41413_2025_475_MOESM5_ESM.tif]

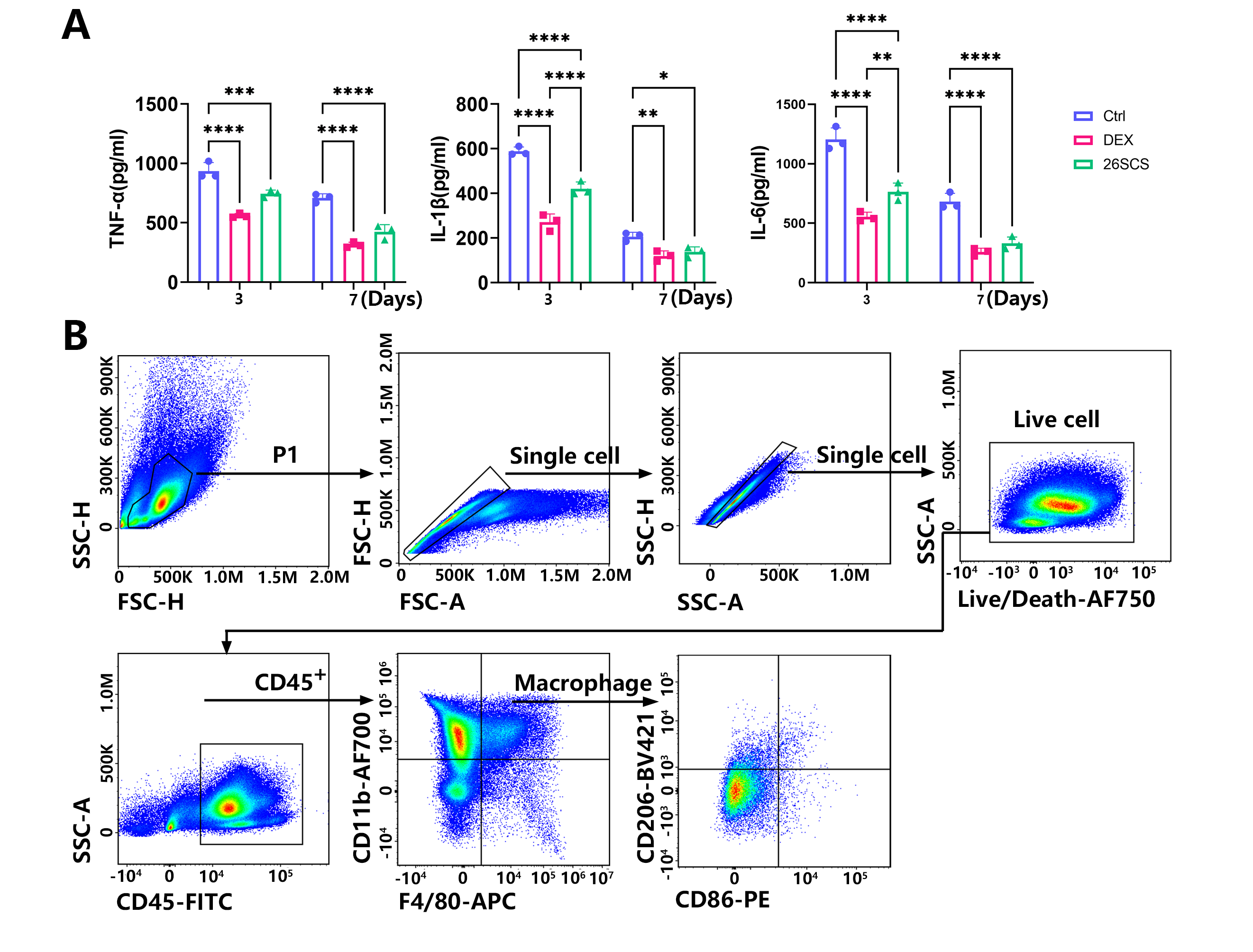

Supplement: Supplementary file 6 — Figure S5 [file 41413_2025_475_MOESM6_ESM.tif]

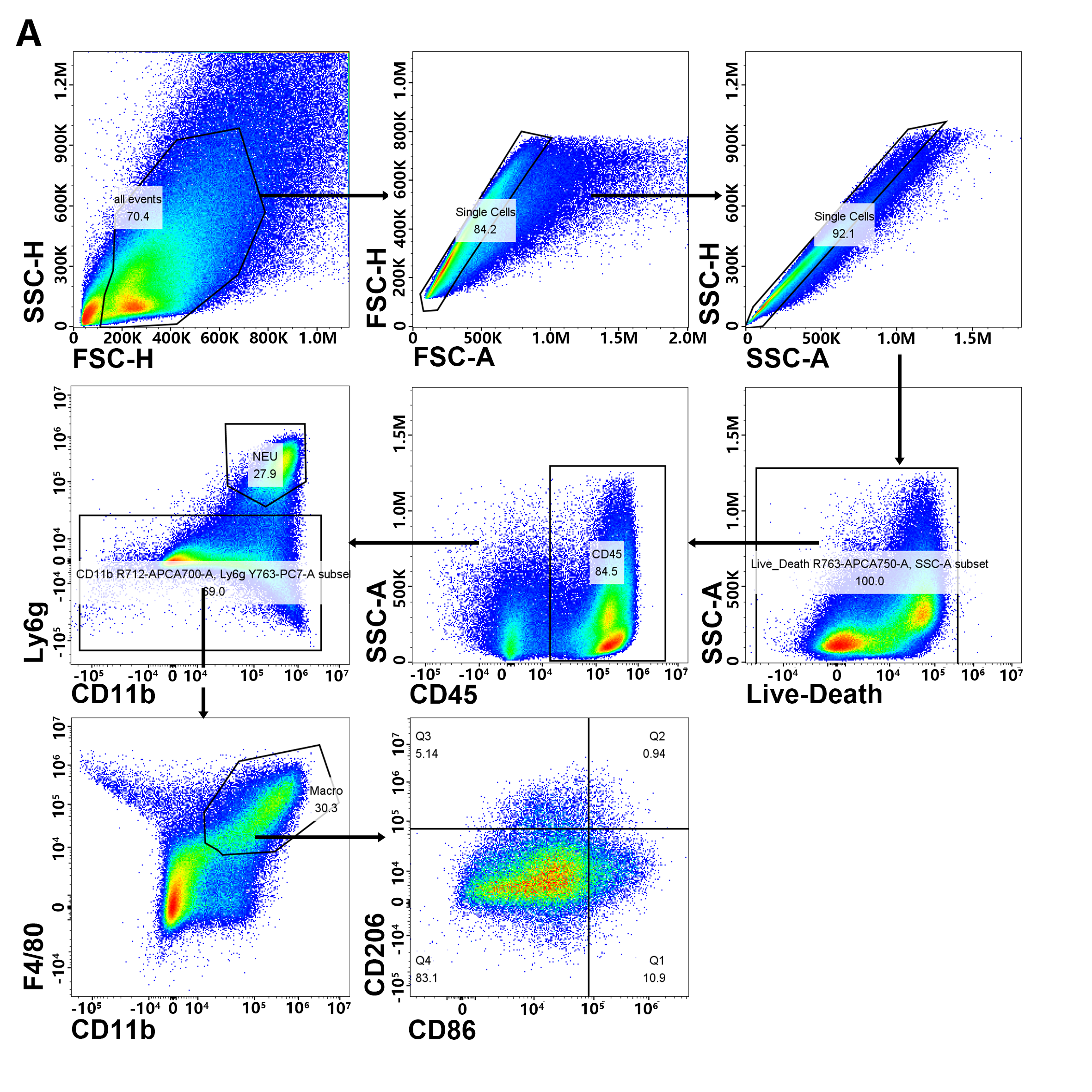

Supplement: Supplementary file 7 — Figure S6 [file 41413_2025_475_MOESM7_ESM.tif]

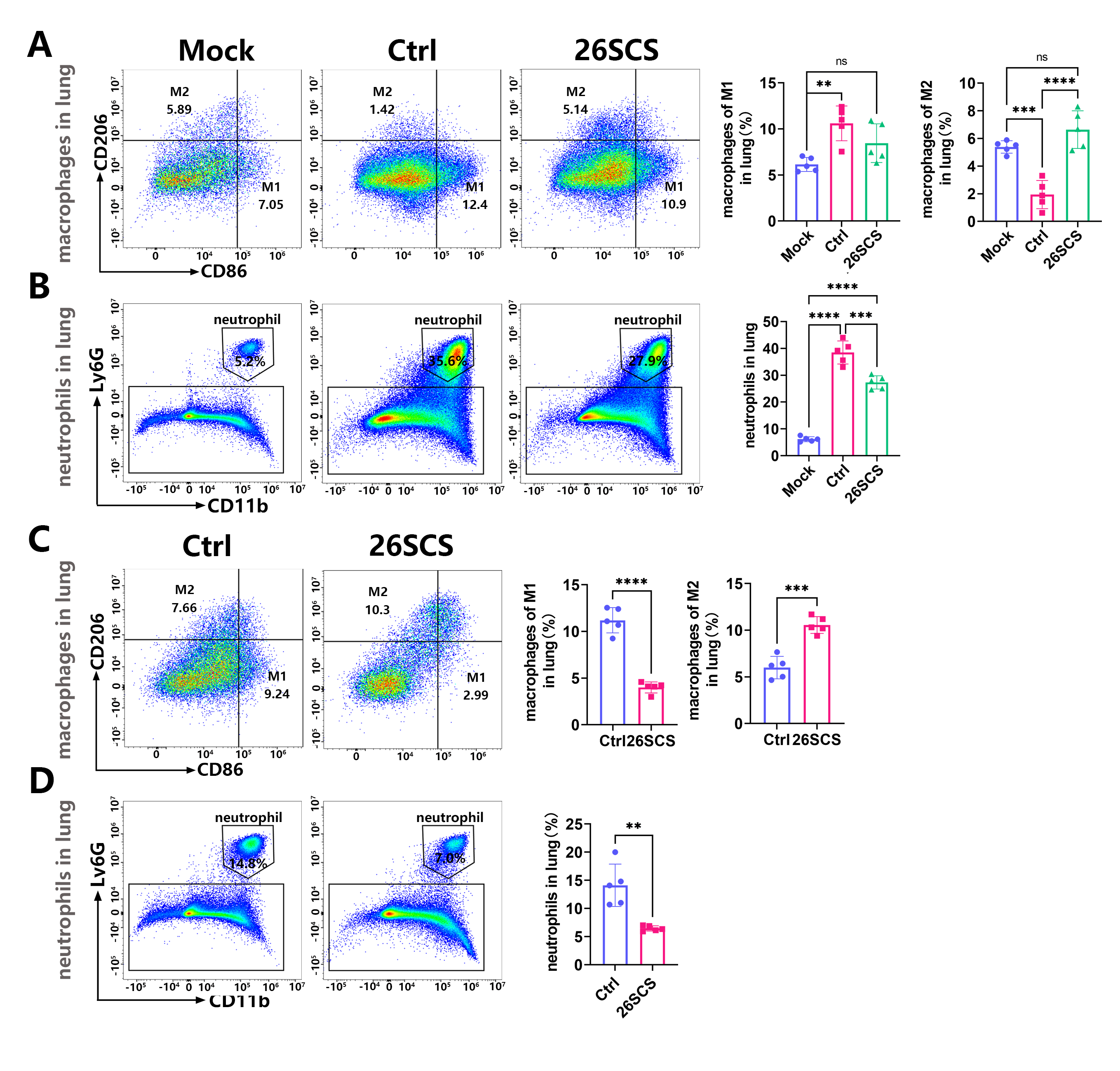

Supplement: Supplementary file 8 — Figure S7 [file 41413_2025_475_MOESM8_ESM.tif]

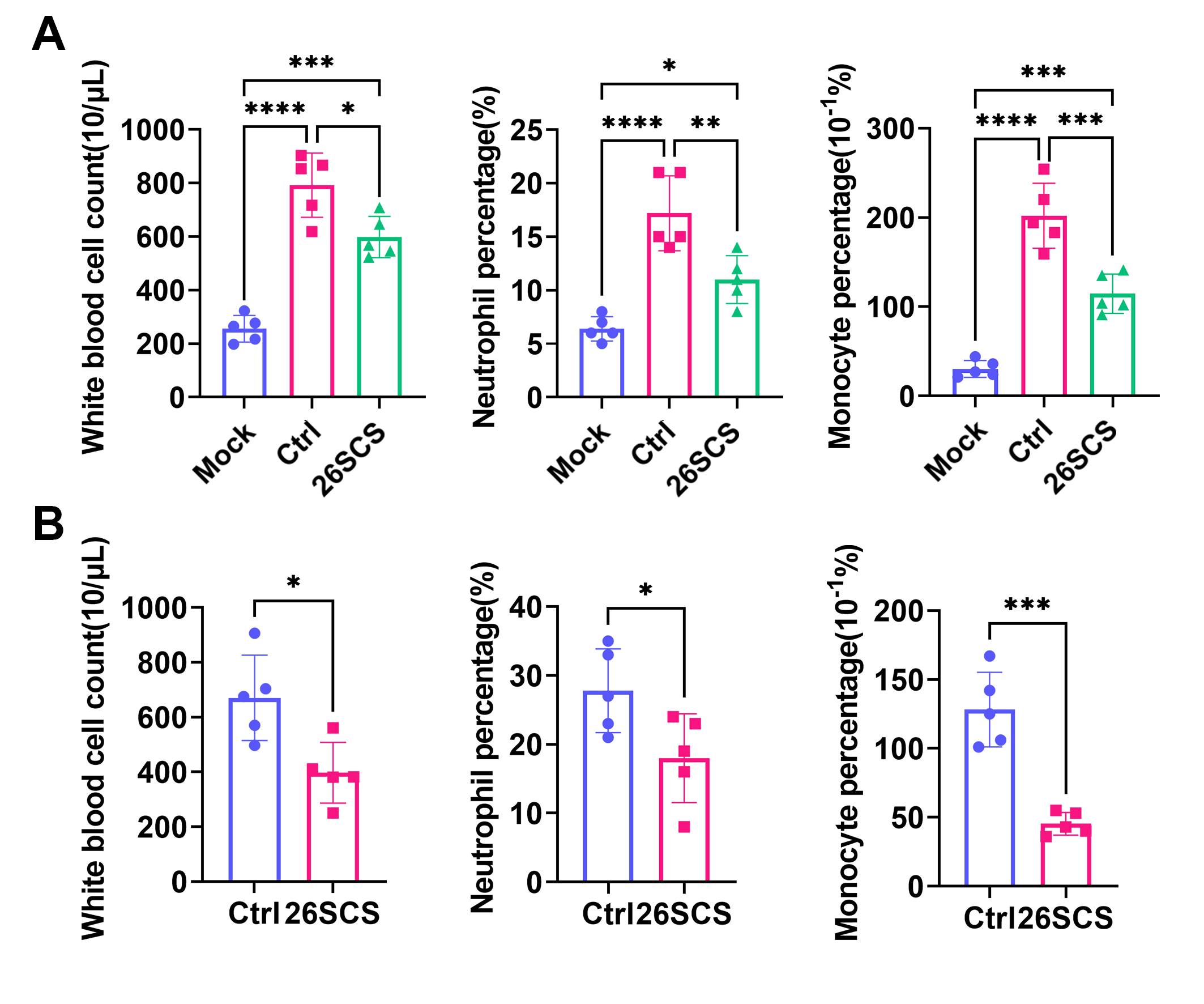

Supplement: Supplementary file 9 — Figure S8 [file 41413_2025_475_MOESM9_ESM.tif]

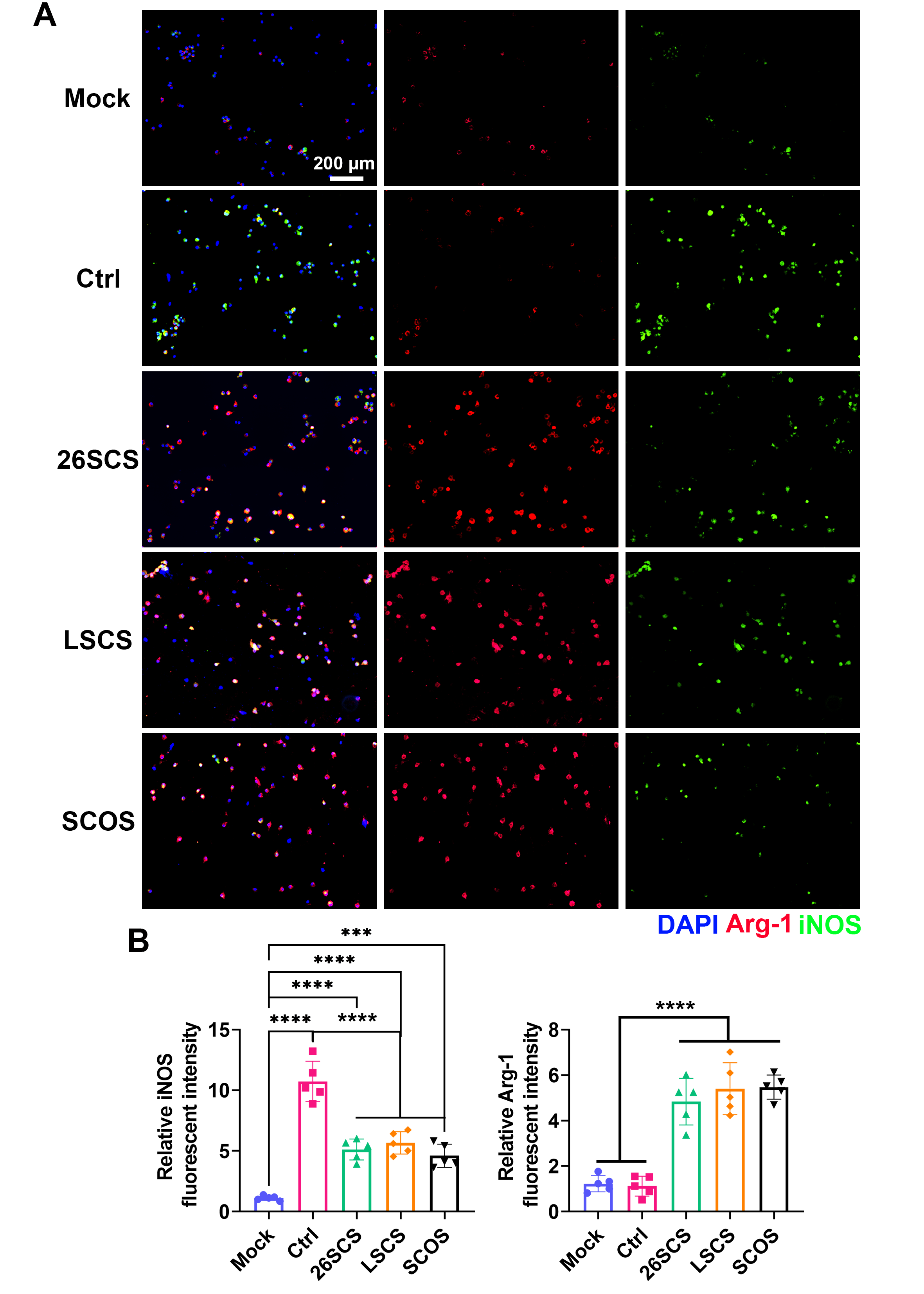

Supplement: Supplementary file 10 — Figure S9 [file 41413_2025_475_MOESM10_ESM.tif]

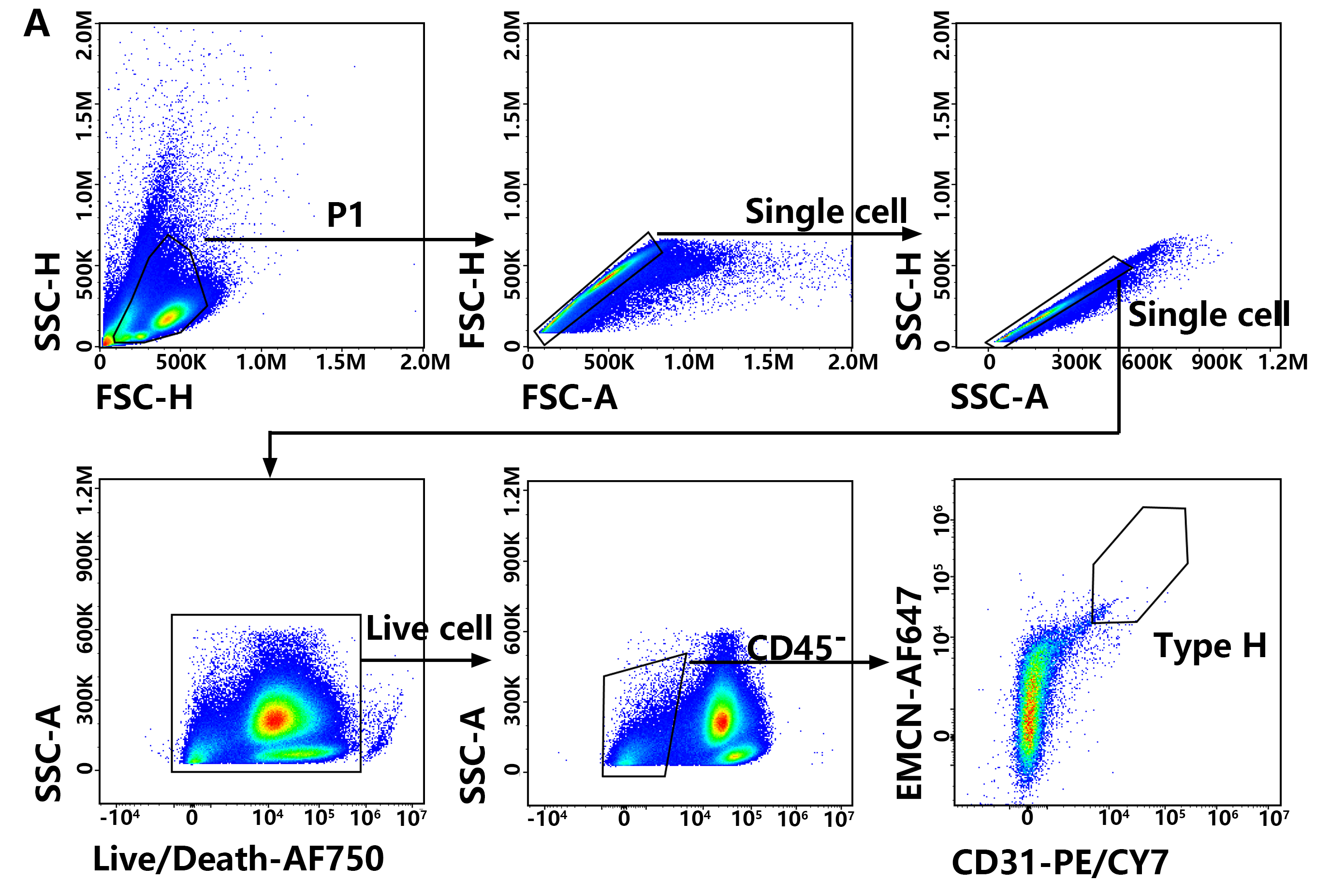

Supplement: Supplementary file 11 — Figure S10 [file 41413_2025_475_MOESM11_ESM.tif]

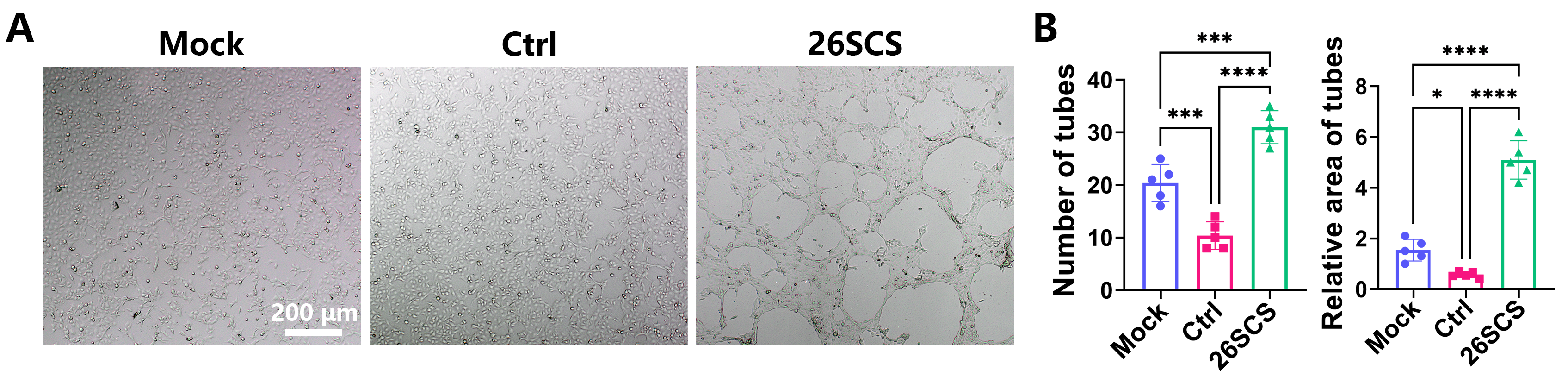

Supplement: Supplementary file 12 — Figure S11 [file 41413_2025_475_MOESM12_ESM.tif]

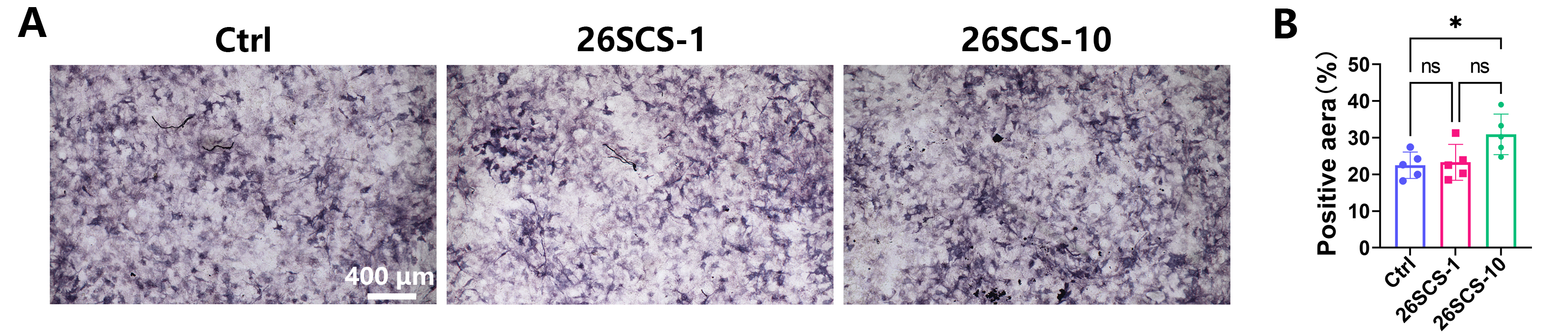

Supplement: Supplementary file 13 — Figure S12 [file 41413_2025_475_MOESM13_ESM.tif]
